# Dosimetrically coupled multiscale tetrahedral mesh models of human liver vasculature: implications for radiopharmaceutical dosimetry of both organ blood and parenchyma

**DOI:** 10.1088/1361-6560/addfa6

**Published:** 2025-06-19

**Authors:** Robert J Dawson, Carlos Huesa-Berral, Lukas M Carter, Chris Beekman, Chansoo Choi, Bangho Shin, Mislav Bobić, Nicolò Cogno, Julia D Withrow, Derek W Jokisch, Alejandro Bertolet, Harald Paganetti, Wesley E Bolch

**Affiliations:** 1 University of Florida, Gainesville, FL, United States of America; 2 Harvard Medical School, Boston, MA, United States of America; 3 Massachusetts General Hospital, Boston, MA, United States of America; 4 Memorial Sloan Kettering Cancer Center, New York, NY, United States of America; 5 Francis Marion University, Florence, SC, United States of America; 6 Oak Ridge National Laboratory, Oak Ridge, TN, United States of America

**Keywords:** computational phantoms, liver vasculature, radiopharmaceutical dosimetry, blood self-dose

## Abstract

**Objective.:**

To develop a computational framework coupling multiscale vascular models of the human liver for improved radiation dosimetry calculations that clearly distinguish the absorbed dose to tissue parenchyma and that to its blood content at all spatial scales. This framework thus addresses limitations of homogeneous blood/tissue organ models in present use in radiopharmaceutical therapy.

**Approach.:**

High-fidelity tetrahedral mesh models of liver vasculature were constructed at two spatial scales. At the macroscale, detailed hepatic arterial, venous, and portal venous networks were generated within reference adult male and female computational phantoms. At the microscale, a classical hexagonal liver lobule model incorporating sinusoids, bile compartments, and cellular components was developed. A mathematical framework was further developed to couple Monte Carlo radiation transport results across these spatial scales, enabling comprehensive dosimetric calculations for radiation dose to both blood and parenchymal tissues.

**Main Results.:**

The coupled model system successfully accounted for the entire blood content of the liver, with approximately 31% represented in macroscale vessels (⩾100 *μ*m diameter) and 69% within microscale structures. Specific absorbed fractions were computed for monoenergetic photons, electrons, and alpha particles, demonstrating reciprocity between blood-to-parenchyma and parenchyma-to-blood crossfire. Reference S-values were computed for 22 therapeutic and 11 diagnostic radionuclides, providing the first comprehensive dataset for blood-specific and parenchyma-specific internal dosimetry calculations in the liver.

**Significance.:**

This work establishes a novel framework for multi-scale radiation transport calculations in vascularized organs, enabling separate tracking of blood and parenchymal tissue doses. The methodology has immediate applications in improving dose calculations for radiopharmaceutical therapies, Y-90 microsphere radioembolization treatment, and analysis of blood dose during external beam radiotherapy. The approach can be readily adapted for other vascularized organs, representing a significant advancement in radiation dosimetry accuracy for both therapeutic and diagnostic applications by fully and independently accounting for organ activity localized within two tissue compartments—organ blood and organ parenchyma.

## 1. Introduction

The mesh-type reference computational phantoms (MRCPs) developed by the International Commission on Radiological Protection (ICRP) and introduced in ICRP Publication 145 represent the current state-of-the-art in computational anthropomorphic phantoms (Kim et al 2020). Despite their advancements, the MRCPs contain only major *inter-organ* veins and arteries, while the organ regions within these phantoms are modeled as homogeneous mixtures of blood and parenchyma, lacking explicit representation of *intra-organ* vasculature. This omission may limit their applicability for dosimetry in scenarios such as radiopharmaceutical therapy and external beam radiotherapy, where detailed vascular modeling could enhance dosimetric accuracy and rigor. For example, in radiopharmaceutical therapy, the time-dependent transfer of the radiopharmaceutical between circulating blood and organ parenchyma, coupled with the short particle ranges and sharp dose-point kernels typical of therapeutic radionuclides, make the spatial distribution of vascular and parenchymal tissues important for accurate dosimetry of these tissues. Namely, refined phantoms incorporating vascular structures with adequate spatial detail will support improved dose calculations by accounting for dose contributions from circulating blood to organ parenchyma and vice versa. This advancement has the potential to yield more reliable dose-effect relationships, enhancing both safety and efficacy in radiopharmaceutical therapy. In addition, in the external-beam radiotherapy setting, the distribution of vasculature impacts the dose received by blood perfusing the organs.

Modeling liver vasculature is a focal point of interest due to the unique characteristics of the liver. The organ is highly perfused with approximately 10% of total body blood content. Furthermore, the liver plays a central role in the metabolism and clearance of radiopharmaceuticals, resulting in significant radiation exposure. The liver is frequently a dose-limiting organ in radiation therapy, where both parenchyma and circulating blood are exposed. Several groups have constructed macroscale models of liver vasculature, primarily for studies regarding the construction of synthetic medical images (Correa-Alfonso et al 2022, Sauer et al 2022, Whitehead et al 2023). Models of liver microstructure have also been developed, either stylistically or based on images from confocal laser microscopy, vascular corrosion casting, or immunohistochemistry (Gulec et al 2010, Hoehme et al 2010, Stenvall et al 2014, Peeters et al 2017). Though several vasculature models have been constructed, at various spatial scales, for use in computational radiation dosimetry studies, none have been paired with an analogous model at a different spatial scale to fully account for all radionuclide sources of blood in the organ.

Fully detailed whole-organ models capturing vascular anatomy down to the capillary level could theoretically be constructed, but such models would far exceed the capabilities of current computing power and memory, even on large supercomputing clusters. The present work therefore proposes a framework for producing dosimetry estimates at all spatial scales by performing independent simulations at different spatial scales and combining the results mathematically.

In this study, high-quality tetrahedral mesh vascular models, at both the macroscale and microscale, have been constructed for both the adult male and adult female MRCPs. For each sex, blood content in the model at each spatial scale was adjusted such that 100% of blood in the liver was explicitly captured. At the macroscale, realistic blood vessel networks for the hepatic arterial, hepatic venous, and portal venous systems were generated within the reference liver surfaces. This represents the first study to create an augmented version of the whole-body MRCPs. At the microscale, liver parenchyma was modeled as the classic hexagonal prism lobule which forms the functional subunit of the liver. Specific absorbed fractions for use in internal dosimetry were computed independently for blood and parenchyma source-target combinations at both spatial scales and mathematically coupled to produce estimates for these quantities across all energy ranges for photons, electrons, and alpha particles. S-values for therapeutic and diagnostic radionuclides of interest were then computed using these composite quantities.

## 2. Materials and methods

### 2.1. Macroscale polygon mesh model

The adult male and female MRCPs each contain a single, large mesh surface modeling the peripheral contour of the whole liver, inclusive of liver parenchyma and blood content. In a previous study, Correa-Alfonso et al (2022) extracted and partitioned these liver volumes into eight segments (each with an appropriate percent volume of the organ) according to the standard Couinaud classification system, and perfused the organs with virtual vasculature using a constrained constructive optimization approach. While this approach utilized known physical and hemodynamical principles, certain deficiencies, such as a lack of vessel curvature and inherent limits in the total volume of blood that could be captured in the network, motivated the development of an updated and more robust vessel centerline generation and meshing approach.

**Figure 1.**
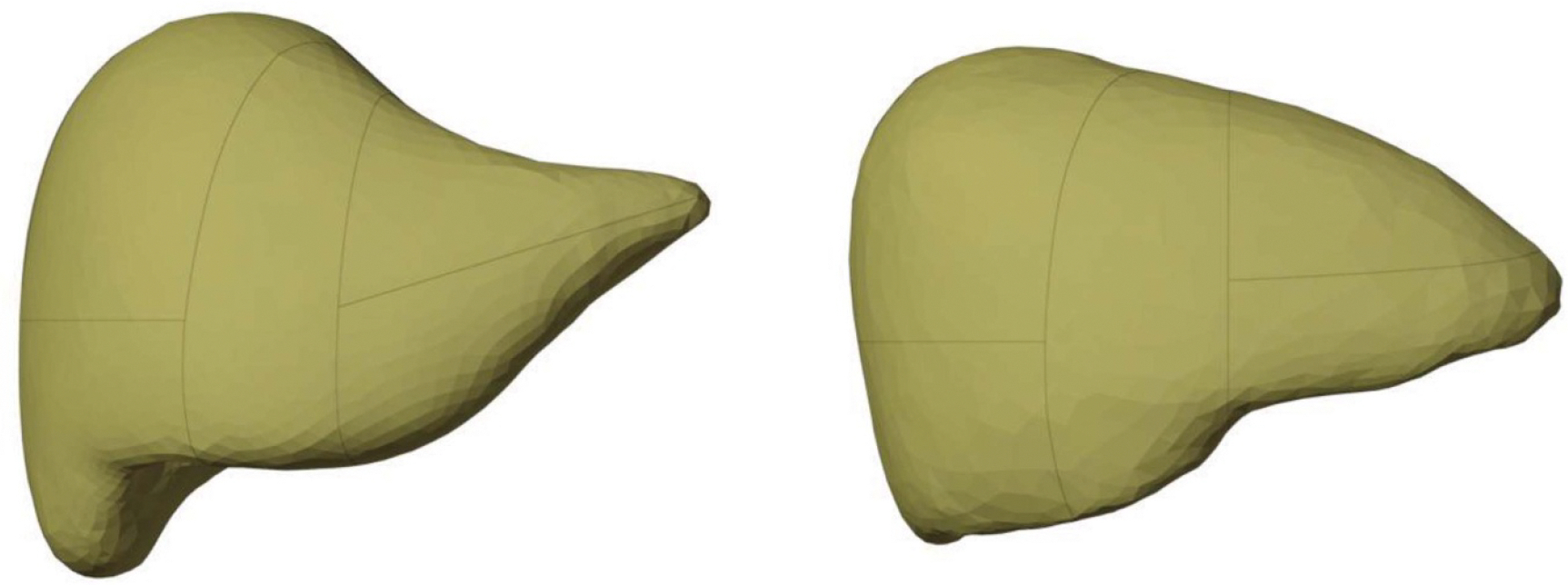
The liver surface of the adult male (left) and adult female (right) MRCP partitioned into the standard eight segments of the Couinaud classification.

Each segment of the partitioned liver volume was voxelized to produce a binary mask with isotropic voxels of size 0.3 mm and entry locations for the major blood vessels into the organ were identified with respect to the organ surface. The partitioned liver segments for both sexes are shown below in figure 1.

The centerline generation algorithm used to generate the mathematical models of the vessel networks was based on the one developed by Whitehead et al (2023). A centerline graph is defined as a collection of nodes (i.e. vertices) and edges (i.e. connected vertices); here, the nodes and edges define vessel segments, with each edge being assigned a radius, such that the segments collectively form a continuous tubular network. The centerline graph was constructed by first randomly generating points within the confines of the organ surface which serve as either terminal endpoints or bifurcation points. Each vessel tree was initialized at the entry points of the major vessel entering the liver (the common hepatic artery, for example) and grew by iteratively adding vessels to the existing tree with prescribed radii for the major input vessels (Zhang et al 2009, Debbaut et al 2011, Geleto et al 2016, Carneiro et al 2019, Tzafriri et al 2022). The growth behavior was constrained by Murray’s law, which selects radii for smaller vessel branches as a function of the larger vessel’s radius, as well as by a volume-minimizing cost function to mimic the natural, energy-conserving angiogenic process (Murray 1926). Other features, such as bifurcation angle optimization, polynomial interpolation for vessel curvature, and robust vessel intersection avoidance, were also implemented in a C++ program (Whitehead et al 2023).

To generate the initial polygon mesh surfaces from the centerline model, a unit cylinder (in mesh format, with radius and height equal to 1 mm and circular faces lying in the x-y plane) was constructed in Blender software such that the center of its base was located at the origin. The centerline model was parsed into individual segments and passed to an in-house C++ program utilizing the Surface Mesh class in the Computational Geometry Algorithms Library (CGAL) version 5.6^7^. To generate each vessel segment (with centerline vector $\vec{v}$), the unit cylinder was copied, scaled in the x and y dimensions by a factor equal to the segment’s target radius, and then transformed according to the equation

(1) $$\overset{⇀}{v}=R\;(\overset{⇀}{u},\theta )\;\overset{ˆ}{k},$$

where $\overset{ˆ}{k}$ is the unit vector along the z-axis, θ is the angle of rotation about a specified unit vector $\overset{⇀}{u}$ that is orthogonal to both the axis of the unit cylinder and the vector defining the segment’s centerline, and $R\;(\overset{⇀}{u},\theta )$ is the Rodriguez rotation matrix:

(2) $$R\;(\overset{⇀}{u},\theta )=\left[\begin{array}{ccc} cos\;\theta +u_{x}^{2}(1-cos\;\theta ) & u_{x}u_{y}(1-cos\;\theta )-u_{z}\;sin\;\theta & u_{x}u_{z}(1-cos\;\theta )+u_{y}\;sin\;\theta \\ u_{y}u_{x}(1-cos\;\theta )+u_{z}\;sin\;\theta & cos\;\theta +u_{y}^{2}(1-cos\;\theta ) & u_{y}u_{z}(1-cos\;\theta )-u_{x}\;sin\;\theta \\ u_{z}u_{x}(1-cos\;\theta )-u_{y}\;sin\;\theta & u_{z}u_{y}(1-cos\;\theta )+u_{x}sin\;\theta & cos\;\theta +u_{z}^{2}(1-cos\;\theta ) \end{array}\right].$$

**Figure 2.**
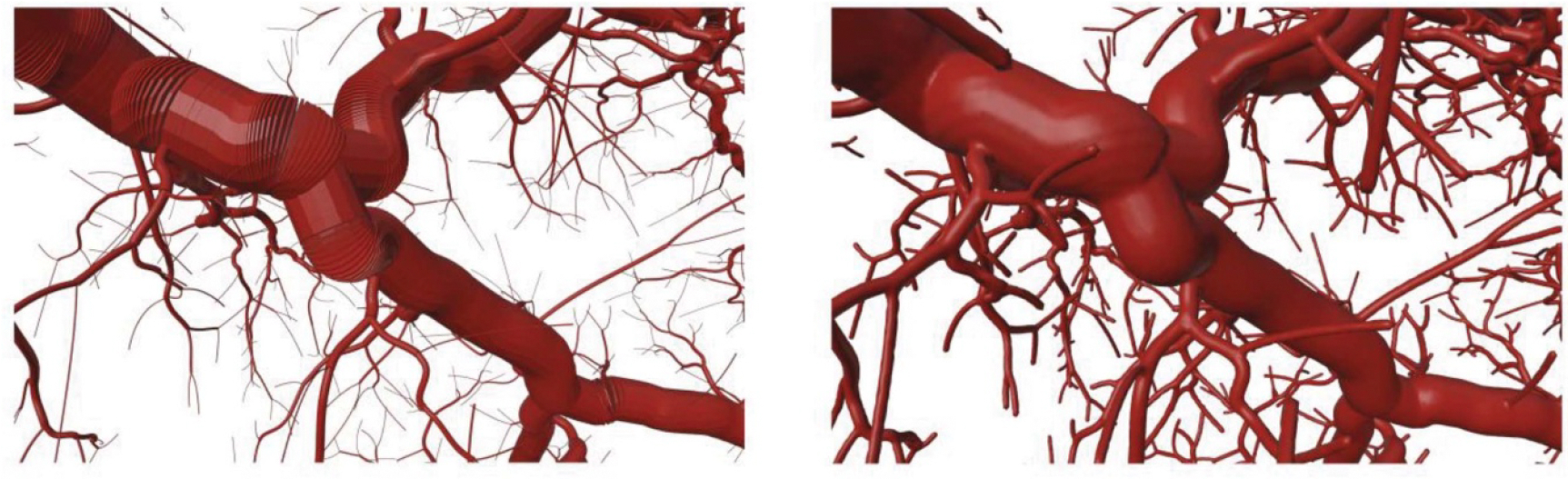
Output from CGAL of transformed cylindrical vessel segments (left) and 2-manifold surface mesh produced via 3D Alpha Wrapping (right). The apparent increase in vessel thickness in the continuous mesh is due to a prescribed offset that is passed to the 3D Alpha Wrapping algorithm and is accounted for by systematically reducing the radii of the input vessel segments by a commensurate thickness.

The transformed segments were then combined into a single polygon mesh object to form a network of disjoint cylinders with mesh intersections at the junctions between segments. The output of this procedure then served as the input to CGAL’s 3D Alpha Wrapping algorithm, producing a 2-manifold mesh surface free of self-intersections and other potential mesh defects. The transition from a collection of individual, cylindrical segments to a continuous mesh surface can be seen in figure 2. Because the 3D Alpha Wrapping procedure adds a compulsory, user-defined nonzero offset to the input geometry, the radii of the initial cylinders were deliberately undersized by a thickness equal to this prescribed offset prior to wrapping. Thus, the final continuous mesh properly represented accurate vessel radii.

**Figure 3.**
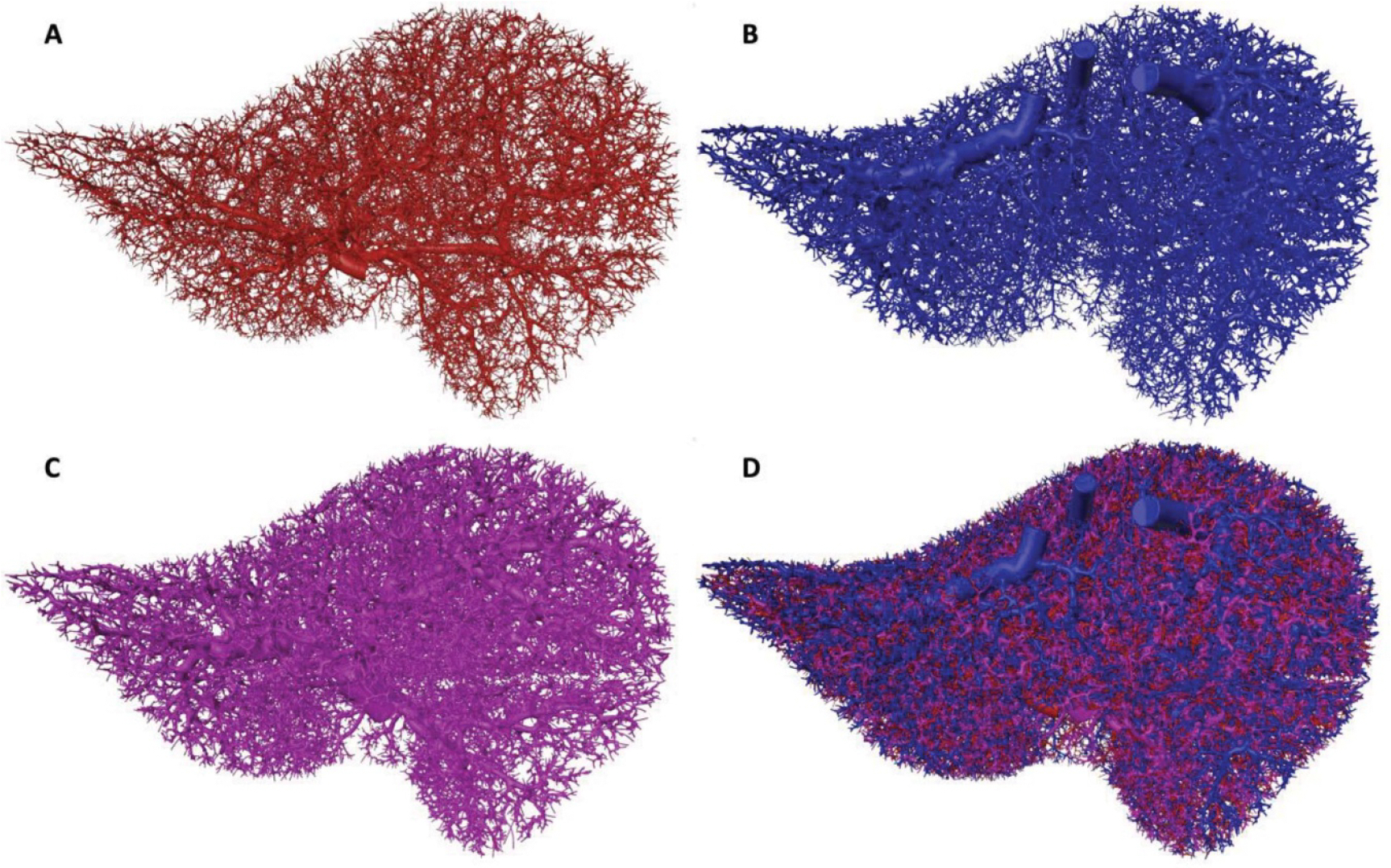
Posterior view of the hepatic arterial (A), hepatic venous (B), hepatic portal venous (C), and combined (D) macroscale vascular networks for the adult male.

**Figure 4.**
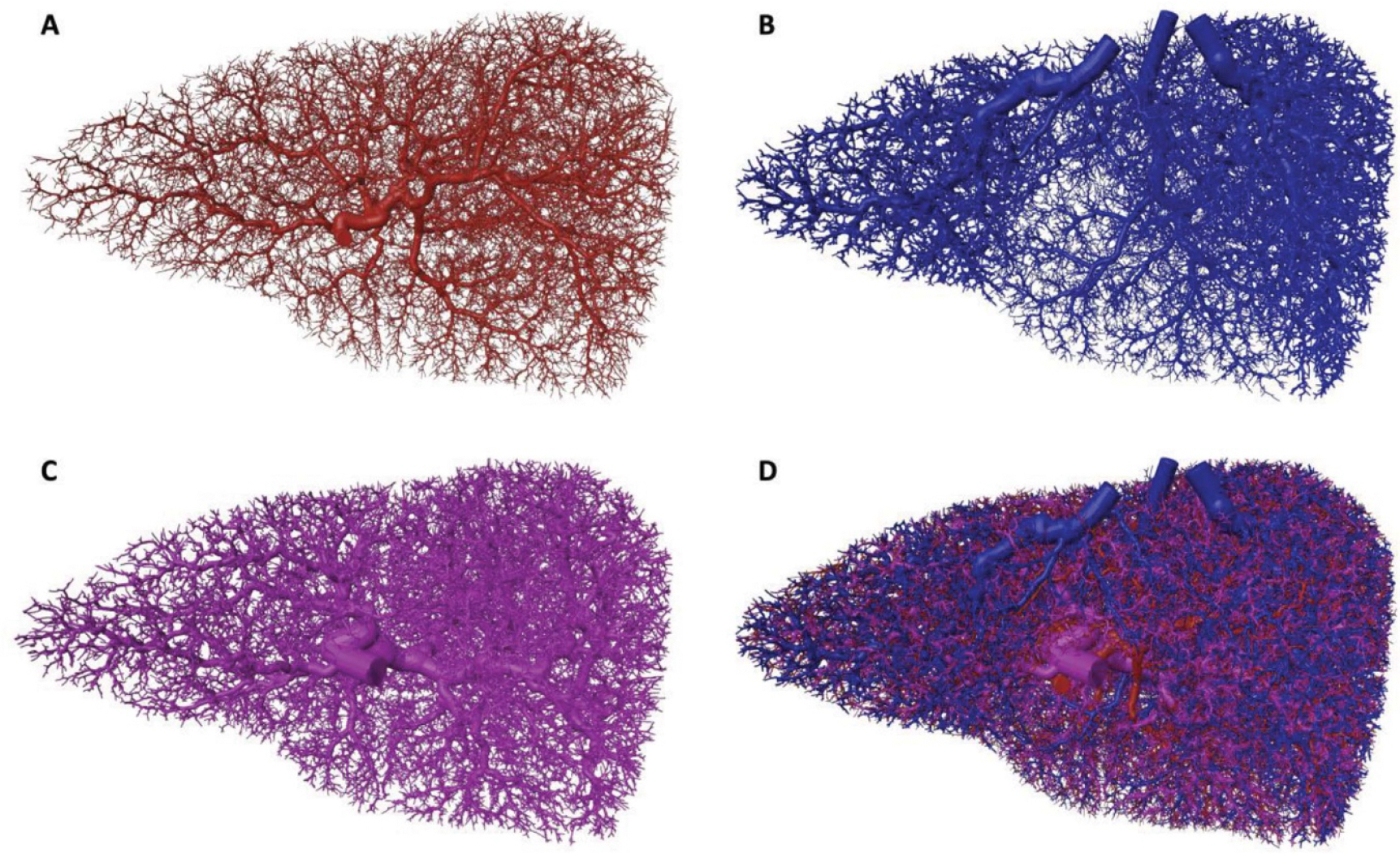
Posterior view of the hepatic arterial (A), hepatic venous (B), hepatic portal venous (C), and combined (D) macroscale vascular networks for the adult female.

For each sex, vascular networks containing 150 000 vessels (50 000 per tree) were constructed. Individual trees and the combined vasculature model for the adult male as shown in figure 3. The minimum vessel radius reached in these models was approximately 100 *μ*m. Vessels in a given tree did not intersect with other vessels in the same tree, nor with vessels in any other tree, with the exception of very small intersections occurring due to numerical precision errors and finite voxel size effects during the centerline generation process; these were resolved by joining the three vessel trees using serial Boolean union operations in CGAL using the *exact predicates*, *exact constructions* kernel. The combined vasculature model for the adult female is shown in figure 4.

To make the models as anatomically accurate as possible, the hepatic arterial and hepatic portal venous trees perfused all eight lobes (I–VIII) of the liver with a single input entry vessel located in the liver hilum. The location, branching patterns, and sizes of hepatic veins entering the liver are highly variable among individuals, but a common configuration with three entry vessels was chosen for modeling purposes: the right hepatic vein, which perfused lobes VI and VII, the middle hepatic vein, which perfused lobes IV, V, and VIII, and the left hepatic vein, which perfused lobes I, II, and III (Sureka et al 2019). The numbers of blood vessels and terminal endpoints in each venous subregion were adjusted to be proportional to the relative volumes of these subregions such that the combined venous system matched the two other vascular systems.

### 2.2. Microscale polygon mesh model

Outside of larger blood vessels of the hepatic arterial, hepatic venous, and portal venous vascular systems, liver parenchyma is generally repetitive and organized into hexagonal subunits called hepatic lobules (Mccuskey 2008, Krishna 2013, Debbaut et al 2014, Lorente et al 2020). A stylized hepatic lobule was constructed using primitive mesh objects and Boolean volume operations in Blender software. The anatomical components of interest and the general architecture of the lobule model were replicated largely based on the mathematical surface/macrobody model proposed by Stenvall et al (2014). These components include bile canaliculi (channels between hepatocyte cords which serve to transport bile within liver parenchyma), a central vein, portal triads (bile ducts, portal arteries, and portal veins), sinusoids, Spaces of Disse (interstitial fluid space surrounding the sinusoids), hepatocytes, and Kupffer cells (Westerhoff and Lamps 2022).

Sinusoids are specialized, fenestrated capillaries within the liver lobule which serve to transport blood between the central vein and portal triads (Torres Rojas et al 2021, Westerhoff and Lamps 2022, Boninsegna et al 2023). These vessels were modeled as a continuous network of straight cylinders (with 16 *μ*m diameter) radiating outward from the central vein toward the edges of the hexagonal prism lobule.

Kupffer cells are macrophages which line the luminal side of the sinusoidal epithelium and, after hepatocytes, are the most numerous cell type within the human liver (Nguyen-Lefebvre and Horuzsko 2015). They also represent the largest population of tissue-residing macrophages in the human body (Dixon et al 2013, Grant and Liberal 2017). Previous studies have established Kupffer cells as important targets for radiation, with dose to these cells inducing liver toxicity (Tello et al 2008, Chen et al 2015). These cells are large and occlude the sinusoidal lumen, making them important geometric features in the context of Monte Carlo simulations. Kupffer cells were procedurally generated within the microscale lobule model by first randomly generating icosahedrons within the region’s bounding box using an in-house Blender Python application programming interface script. These randomly positioned cells were then refined by computing their volume intersection with the previously constructed sinusoids mesh, thus ensuring that all Kupffer cells were contained within the sinusoids. This process was iteratively repeated until the target volume of cells, across the entire lobule, was reached. This target volume, along with the spatial dimensions of all anatomical structures modeled (with the exception of the sinusoids) were replicated from Stenvall et al (2014). The sinusoids were oversized compared to the reference in order to capture more overall relative blood content in the microscale model.

**Figure 5.**
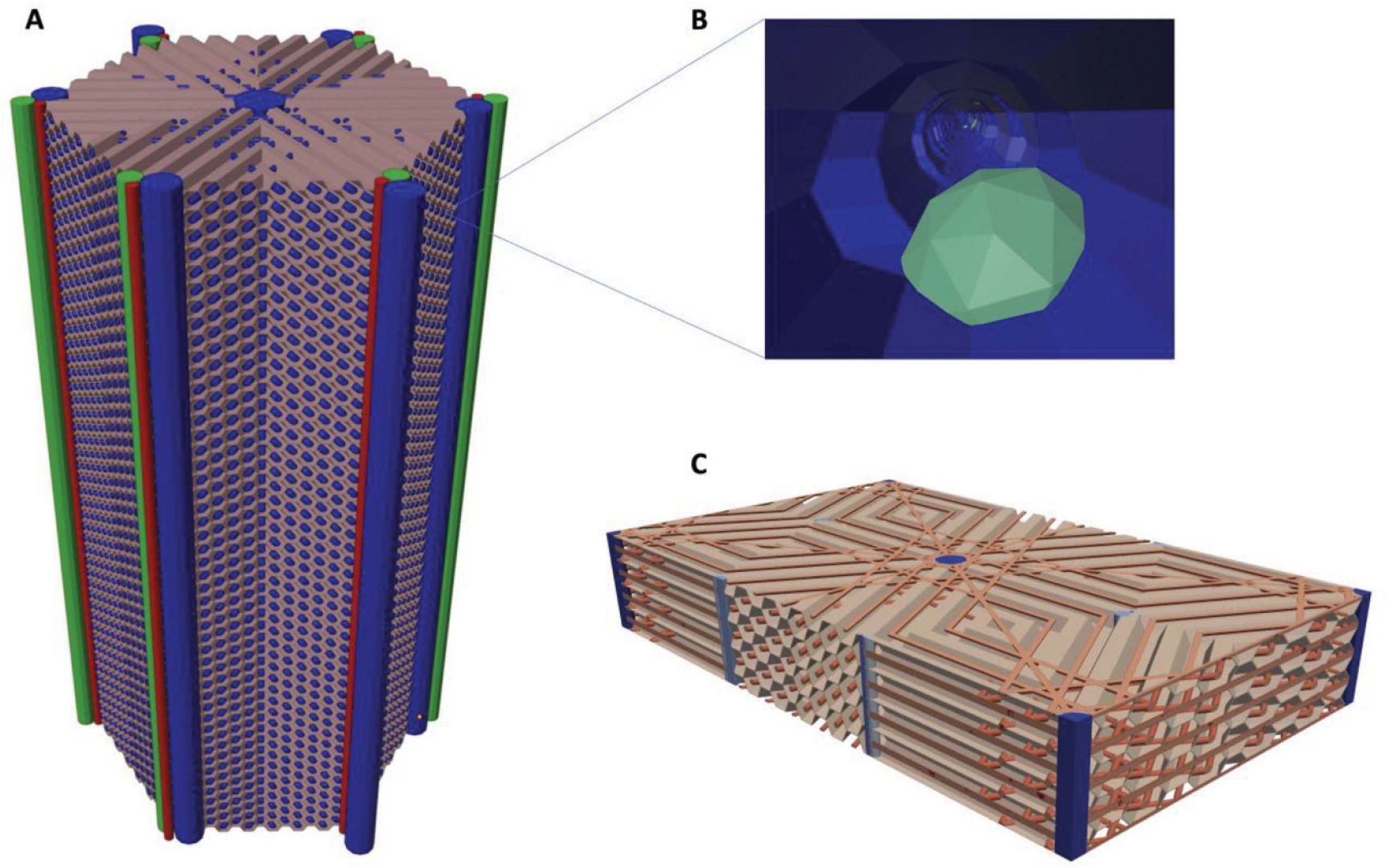
Microscale liver lobule model includes hepatic arteries and veins, portal veins, central vein, bile ducts, bile canaliculi, blood sinusoids, space of Disse, and Kupffer cells. (A) Hexagonal lobule model in polygon mesh format. (B) Kupffer cells adhering to the (luminal side of) epithelia of the sinusoid network. (C) Refined rectangular prism lobule model in tetrahedral mesh format (hepatocyte-labeled tetrahedral which fill the empty space in the model are omitted for clarity).

The microscale model was created in polygon mesh format in the shape of a hexagonal prism. To make it more geometrically amenable to reflective boundary conditions in the Monte Carlo radiation transport simulations, a repeatable, rectangular prism representing this anatomy was created using the hexagonal lobule. The original lobule was duplicated and translated six times such that each duplicate was located adjacent to the original central lobule. This combined model was then cropped using mesh Boolean operations to form a rectangular prism-shaped model with symmetric boundaries, such that any particles leaving the region and reflected inward would see mirrored geometry. This procedure preserved all targeted relative mass fractions among the represented tissues. Renderings of the hexagonal lobule and the refined rectangular prism lobule are shown in figure 5.

**Figure 6.**
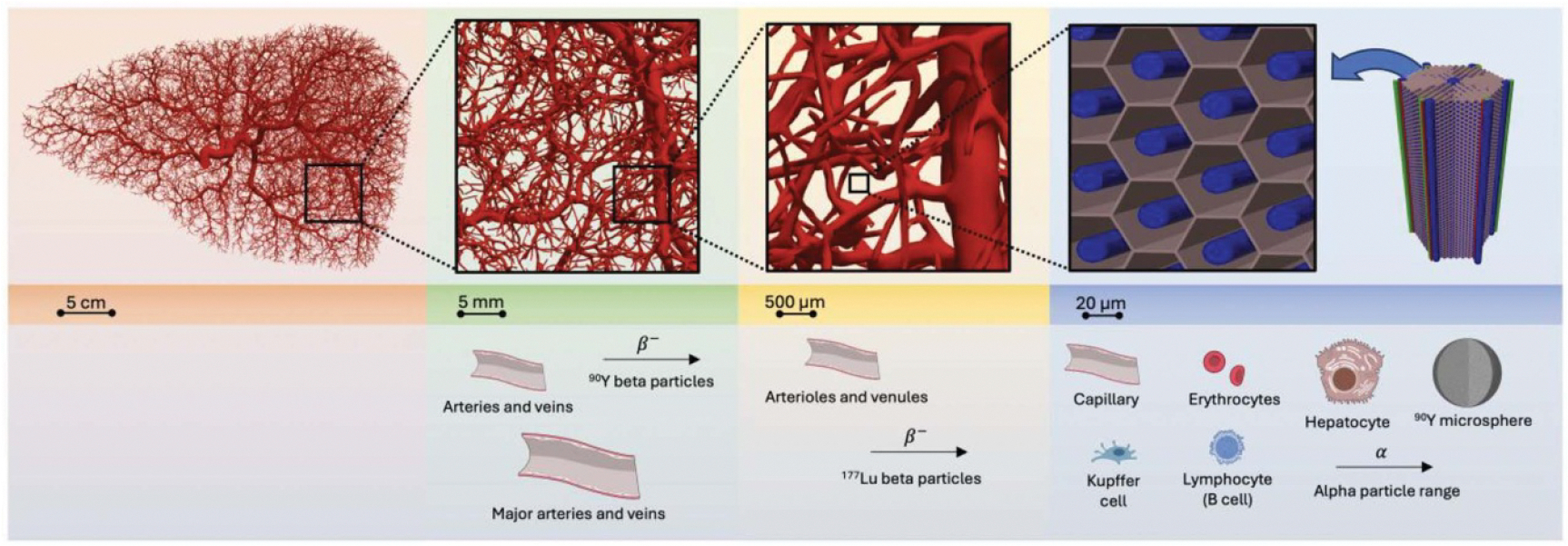
Overview of multiscale liver vasculature model. Shown, from left to right, are decreasing spatial scales and the associated cell types, radiations, and other objects which are relevant at that scale. Diagrams representing cell types and blood vessels were taken from the NIH BioArt Source.^8^

An overview of the coupled vascular models, with associated cell types and radiations relevant at different spatial scales, is shown in figure 6.

### 2.3. Derivation of material compositions and mass targets

Of note, approximately 60% of the blood in the liver is contained in the sinusoids (Eipel et al 2010). Thus, using the overall mass of blood in the liver as defined in ICRP Publication 145, and the ratio of sinusoid blood to other blood compartments in the lobule (central vein, portal arteries, portal veins), a target blood mass in the macroscale vessel network was calculated (Kim et al 2020). The number of terminal endpoints (and therefore number of vessels) in the macroscale models was kept as a free parameter and iteratively tuned until the target volume was achieved, thereby accounting for 100% of liver blood volume in the liver across both the macroscale and microscale domains. Of note, the volume of blood captured by the combined macroscale model accounts for 31.5% of blood in the liver in the adult male, and 30.6% in the adult female, with the balance accounted for in the microscale lobule structures which implicitly fill the macroscale parenchyma volume. Relevant masses for the coupled liver vasculature models can be found in table 1.

The determination of material compositions in the macroscale models was straightforward, with direct elemental mass fractions taken from ICRP Publication 145 for blood and homogenized liver (Kim et al 2020). The composition of liver parenchyma (the regions outside of the explicitly modeled blood vessels in the macroscale models) was determined by calculating the absolute quantities of each element in the homogenized liver and in the explicitly modeled blood vessels, finding the difference for each element, and renormalizing these quantities based on the macroscopic parenchyma mass. This ensured that the overall liver composition, if homogenized, was of the same material as the homogenized liver medium in the MRCP.

The tissue elemental compositions in the microscale model were determined in a similar manner. Blood-containing regions (central vein, portal arteries, portal veins, and sinusoids) were assigned elemental compositions equivalent to blood in the macroscale model, while bile-containing regions (bile ducts, bile canaliculi) and the interstitial fluid-containing Space of Disse were assigned the blood plasma and lymph fluid material reported in ICRU Report 46 (International Commission on Radiation Units and Measurements 1992). Finally, Kupffer cells and hepatocytes were defined to have the same material composition; elemental mass fractions for this material were kept as a set of free parameters with which to match the overall material composition of the entire model to that of the liver parenchyma material defined for the macroscale models. The distributions of masses and material compositions for the microscale model can be found in tables 2 and 3, respectively.

### 2.4. Tetrahedralization of polygon mesh models

The macroscale vascular tree models were seamlessly integrated into the adult MRCPs. This was done by computing the Boolean intersection of the combined vasculature mesh with the liver surface, with the result replacing the single-region, homogenized liver in the whole-body MRCP. Finally, the newly augmented whole-body phantom was tetrahedralized (space-filled) using the POLY2TET program in preparation for Monte Carlo radiation transport simulations (Han et al 2020).

For the microscale model, small geometric features (on the order of microns) led to numerical precision issues which prevented POLY2TET or TetGen from ever terminating (Si 2015, Han et al 2020). Instead, a custom program utilizing the 3D Mesh Generation package in CGAL was created and used to tetrahedralize the model with prescribable tetrahedral mesh criteria such that all geometric features were well represented.

### 2.5. Monte Carlo radiation transport simulations—macroscale

Radiation transport simulations were performed in the Particle and Heavy Ion Transport code System (PHITS) version 3.24 (Sato et al 2018, 2023). All simulations were computed using the University of Florida HiPerGator computing cluster. In both the adult male and adult female macroscale models, monoenergetic sources were instantiated in PHITS using the ‘tetreg’ (s-type = 24) source mode. Details related to the Monte Carlo simulations (for both the macroscale and microscale models) can be found in table 4. Two sets of simulations were performed for each sex: one set with the source uniformly distributed in the blood vessels, and the other with the source uniformly distributed in the region of the liver outside of the blood vessels.

### 2.6. Monte Carlo radiation transport simulations—microscale

In the microscale model, the presence of near-zero volume tetrahedra caused source initialization errors using the ‘tetreg’ (s-type = 24) source mode in PHITS. To circumvent this issue, a custom C++ program was written to sample points directly from individual tetrahedra in the phantom’s node and element files using random number sampling within a barycentric coordinate system. To do this, the volumes of all tetrahedra were computed and then used to weight the probability of selecting a given tetrahedron. For a tetrahedron with vertices at points $A,\;B,\;C,D\in R^{3}$, its volume can be computed by calculating the determinant

(3) $$V=\frac{1}{6}\left|det\left(\begin{array}{cccc} x_{A} & y_{A} & z_{A} & 1\\ x_{B} & y_{B} & z_{B} & 1\\ x_{C} & y_{C} & z_{C} & 1\\ x_{D} & y_{D} & z_{D} & 1 \end{array}\right)\right|.$$

The set of tetrahedra volumes were then normalized by the maximum volume in the set to create acceptance/rejection thresholds which were compared in each iteration to the random number sampled from a uniform distribution between 0 and 1.

Once a tetrahedron was selected for sampling, the weights for four random coefficients in the barycentric system, $\delta_{1},\;\delta_{2},\;\delta_{3},\;\delta_{4}\in [0,\;1]$, were generated such that $\delta_{1}+\delta_{2}+\delta_{3}+\delta_{4}=1$. This was done by first sampling four random numbers $u_{1},\;u_{2},\;u_{3},\;u_{4}$ from the range [0, 1] and calculating

(4) $$\delta_{i}=\frac{u_{i}}{u_{1}\;+\;u_{2}\;+\;u_{3}\;+\;u_{4}}$$

for i∈{1,2,3,4}. The Cartesian coordinates of the random point P were then computed using these barycentric coordinates according to the equation

(5) $$P=\left(\begin{array}{c} x_{P}\\ y_{P}\\ z_{P} \end{array}\right)=\left(\begin{array}{c} \delta_{1}x_{A}+\delta_{2}x_{B}+\delta_{3}x_{C}+\delta_{4}x_{D}\\ \delta_{1}y_{A}+\delta_{2}y_{B}+\delta_{3}y_{C}+\delta_{4}y_{D}\\ \delta_{1}z_{A}+\delta_{2}z_{B}+\delta_{3}z_{C}+\delta_{4}z_{D} \end{array}\right).$$

This procedure was performed for each of the nine subregions (tissue components) within the model, with 10^8^ points randomly generated for each subregion. These randomly generated points were then imported into PHITS as a dump file source. Within PHITS, the bounding box of the model was introduced into the simulations as a reflective surface. This approach served to represent the microscale lobule model as infinitely filling all 3D space. The following sections describe the way in which the results from this infinite filling approach are reconciled and combined with the results from the Monte Carlo radiation transport results in the macroscale model.

### 2.7. Dosimetric coupling of macroscale and microscale models—mathematical formalism

At each spatial scale—macroscale and microscale—the vascularized liver tissue model is partitioned into spatial domains containing purely blood, purely parenchyma, or both. At the *macro*scale, the domains are the following: ${\mathrm{LB}}_{M}$, denoting ‘pure’ blood in the branches of the hepatic vascular network with radii ⩾~100 *μ*m (i.e. the ‘macroscale polygon mesh model’ of §2.1); and ${LCT}_{M}$, denoting the surrounding composite tissue region comprising both liver parenchyma and blood in vessels of radii <~100 *μ*m (e.g. arterioles, venules, and capillaries). At the *micro*scale, the domains are the microscale blood, ${LB}_{\mu }$, and microscale parenchyma, ${LP}_{\mu }$, which partition ${LCT}_{M}$ into pure blood and parenchyma components. It is important to note that in this formulation, the ${LB}_{\mu }$ and ${LP}_{\mu }$ domains refer to the entire extent of the blood and parenchyma microscale contributions throughout the liver (outside of the macroscale blood vessels), rather than in a single hepatic lobule.

Thus, only three of these domains are spatially disjoint (i.e. non-overlapping): blood within the macroscale vessels, ${LB}_{M}$, blood within the microscale vessels, ${LB}_{\mu }$, and liver parenchyma exclusive of blood, ${LP}_{\mu }$. Let LB represent the total blood and LP the bloodless parenchyma in the liver. Then these spatial domains are related as follows:

(6) $$LB={LB}_{M}\cup {LB}_{\mu },$$

(7) $${LCT}_{M}={LP}_{\mu }\cup {LB}_{\mu },$$

and

(8) $$LP={LP}_{\mu }.$$

The final statement follows from the fact that parenchyma is modeled exclusive of blood only and fully at the microscale. Given the equivalency of LP and ${LP}_{\mu }$, only LP will be used for consistency.

Finally, the entire liver domain, L, can then be simply expressed as the union

(9) $$L={LB}_{M}\cup {LB}_{\mu }\cup LP.$$

The definition of LP has been left as the collection of all tissue components in the microscale liver lobule which lack blood: bile ducts, bile canaliculi, space of Disse, Kupffer cells, and hepatocytes. Similarly, the definition of ${LB}_{\mu }$ is all those which contain only blood: central vein, portal arteries, portal veins, and sinusoids. In this way, blood and non-blood regions have been completely partitioned and thus there are no components which are a homogenized mixture of blood and any other tissue type.

In the following series of mathematical arguments, let $m_{i}$ be the mass of the *i*th tissue component, $r_{T}$ the target regions, $r_{S}$ the source regions, and $\phi_{j}\left(r_{T}\leftarrow r_{S}\right)$ the absorbed fraction of energy derived from Monte Carlo radiation transport at the *j*th spatial scale (macro, M, or micro, μ). Overall absorbed fractions (composites of both spatial scales) are denoted without subscripts as $\phi \;\left(r_{T}\leftarrow r_{S}\right)$.

### 2.8. Dosimetric coupling of macroscale and microscale models—blood-parenchyma crossfire

The following notation and internal dosimetric quantities derive from the MIRD formalism (Bolch et al 2009, MIRD Committee of the SNMMI 2022). To calculate the absorbed fraction from blood to parenchyma, ϕ(LP←LB), the quantities $\phi \;\left(LP\leftarrow {LB}_{M}\right)$ and $\phi \;\left(LP\leftarrow {LB}_{\mu }\right)$ must first be computed to account for parenchyma dose contributions from both macroscale and microscale blood sources. For the first case,

(10) $$\phi \;\left(LP\leftarrow {LB}_{M}\right)=\phi_{M}\left({LCT}_{M}\leftarrow {LB}_{M}\right)\times \frac{m_{LP}}{m_{{LCT}_{M}}}.$$

Scaling by the final term in this equation is necessary since LP is a proper subdomain of ${LCT}_{M}$. This approach also assumes that the densities and elemental compositions of the components of ${LCT}_{M}$ (i.e. blood and parenchymal tissue) are approximately the same.

For the second case,

(11) $$\phi \;\left(LP\leftarrow {LB}_{\mu }\right)=\phi_{\mu }\left(LP\leftarrow {LB}_{\mu }\right)\times \phi_{M}\left({LCT}_{M}\leftarrow {LCT}_{M}\right).$$

The second term on the right side of this equation accounts for the loss of energy from the ${LCT}_{M}$ region at the macroscale, which would otherwise be neglected in the infinite filling conditions of the microscale transport simulations. To produce a final estimate of the absorbed fraction of energy emitted from the liver blood and deposited in the parenchyma, the previous absorbed fraction quantities are source-mass-weighted and combined:

(12) $$\phi \;(LP\leftarrow LB)=\phi \;\left(LP\leftarrow {LB}_{M}\right)\times \frac{m_{{LB}_{M}}}{m_{LB}}+\phi \;\left(LP\leftarrow {LB}_{\mu }\right)\times \frac{m_{{LB}_{\mu }}}{m_{LB}}.$$

The specific absorbed fraction is then

(13) $$\Phi \;(LP\leftarrow LB)=\frac{\phi \;(LP\;\leftarrow \;LB)}{m_{LP}}.$$

### 2.9. Dosimetric coupling of macroscale and microscale models—parenchyma-blood crossfire

To calculate the absorbed fraction for parenchyma-to-blood crossfire, ϕ(LB←LP), the quantities $\phi \;\left({LB}_{\mu }\leftarrow LP\right)$ and $\phi \;\left({LB}_{M}\leftarrow LP\right)$ are computed as

(14) $$\phi \;\left({LB}_{\mu }\leftarrow LP\right)=\phi_{\mu }\left({LB}_{\mu }\leftarrow LP\right)\times \phi_{M}\left({LCT}_{M}\leftarrow {LCT}_{M}\right)$$

and

(15) $$\phi \;\left({LB}_{M}\leftarrow LP\right)=\phi_{M}\left({LB}_{M}\leftarrow {LCT}_{M}\right).$$

Because the source geometries in both cases are identical while the targets are spatially disjoint, the contributions to the two targets comprising the total liver blood can be combined according to the equation

(16) $$\phi \;(LB\leftarrow LP)=\phi \;\left({LB}_{\mu }\leftarrow LP\right)+\phi \;\left({LB}_{M}\leftarrow LP\right)$$

which yields the final equation for the absorbed fraction:

(17) $$\phi \;(LB\leftarrow LP)=\phi_{\mu }\left({LB}_{\mu }\leftarrow LP\right)\times \phi_{M}\left({LCT}_{M}\leftarrow {LCT}_{M}\right)+\phi_{M}\left({LB}_{M}\leftarrow {LCT}_{M}\right).$$

The specific absorbed fraction is then

(18) $$\Phi \;(LB\leftarrow LP)=\frac{\phi \;(LB\;\leftarrow \;LP)}{m_{LB}}.$$

### 2.10. Dosimetric coupling of macroscale and microscale models—parenchyma self-dose

The fraction of energy emitted by the liver parenchyma and absorbed by the parenchyma follows directly from simulations at the microscale which are adjusted by the fraction of energy escaping from the $LP_{M}$ domain at the macroscale:

(19) $$\phi \;(LP\leftarrow LP)=\phi_{\mu }(LP\leftarrow LP)\times \phi_{M}\left({LCT}_{M}\leftarrow {LCT}_{M}\right).$$

The specific absorbed fraction is then

(20) $$\Phi \left(LP\leftarrow LP\right)=\frac{\phi \;\left(LP\;\leftarrow \;LP\right)}{m_{LP}}.$$

### 2.11. Dosimetric coupling of macroscale and microscale models—blood self-dose

As in the blood-parenchyma crossfire case, blood self-dose has contributions from both macroscale and microscale blood sources. The absorbed fraction ϕ(LB←LB) can thus be derived by first calculating the quantities $\phi \;\left(LB\leftarrow {LB}_{M}\right)$ and $\phi \;\left(LB\leftarrow {LB}_{\mu }\right)$.

The macroscale contribution to blood dose can be expanded as

(21) $$\phi \;\left(LB\leftarrow {LB}_{M}\right)=\phi \;\left({LB}_{M}\leftarrow {LB}_{M}\right)+\phi \;\left({LB}_{\mu }\leftarrow {LB}_{M}\right).$$

The first term on the right side of this equation, $\phi \;\left({LB}_{M}\leftarrow {LB}_{M}\right)$, comes directly from simulations with the macroscale model; that is, $\phi \;\left({LB}_{M}\leftarrow {LB}_{M}\right)=\phi_{M}\left({LB}_{M}\leftarrow {LB}_{M}\right)$. The second term can be written as

(22) $$\phi \;\left({LB}_{\mu }\leftarrow {LB}_{M}\right)=\phi_{M}\left({LCT}_{M}\leftarrow {LB}_{M}\right)\times \frac{m_{{LB}_{\mu }}}{m_{{LCT}_{M}}},$$

which yields

(23) $$\phi \;\left(LB\leftarrow {LB}_{M}\right)=\phi_{M}\left({LB}_{M}\leftarrow {LB}_{M}\right)+\phi_{M}\left({LCT}_{M}\leftarrow {LB}_{M}\right)\times \frac{m_{{LB}_{\mu }}}{m_{{LCT}_{M}}}.$$

For the microscale contributions to blood self-dose, one can similarly write

(24) $$\phi \;\left(LB\leftarrow {LB}_{\mu }\right)=\phi \;\left({LB}_{M}\leftarrow {LB}_{\mu }\right)+\phi \;\left({LB}_{\mu }\leftarrow {LB}_{\mu }\right),$$

where

(25) $$\phi \;\left({LB}_{M}\leftarrow {LB}_{\mu }\right)=\phi_{M}\left({LB}_{M}\leftarrow {LCT}_{M}\right)\times \frac{m_{{LB}_{\mu }}}{m_{{LCT}_{M}}}$$

and

(26) $$\phi \;\left({LB}_{\mu }\leftarrow {LB}_{\mu }\right)=\phi_{\mu }\left({LB}_{\mu }\leftarrow {LB}_{\mu }\right)\times \phi_{M}\left({LCT}_{M}\leftarrow {LCT}_{M}\right),$$

which are then combined to produce the expression

(27) $$\phi \;\left(LB\leftarrow {LB}_{\mu }\right)=\phi_{M}\left({LB}_{M}\leftarrow {LCT}_{M}\right)\times \frac{m_{{LB}_{\mu }}}{m_{{LCT}_{M}}}+\phi_{\mu }\left({LB}_{\mu }\leftarrow {LB}_{\mu }\right)\times \phi_{M}\left({LCT}_{M}\leftarrow {LCT}_{M}\right).$$

Finally, overall blood self-dose is calculated by combining the absorbed fractions $\phi \;\left(LB\leftarrow {LB}_{M}\right)$ and $\phi \;\left(LB\leftarrow {LB}_{\mu }\right)$ according to the following equation:

(28) $$\phi \;(LB\leftarrow LB)=\phi \;\left(LB\leftarrow {LB}_{M}\right)\times \frac{m_{{LB}_{M}}}{m_{LB}}+\phi \;\left(LB\leftarrow {LB}_{\mu }\right)\times \frac{m_{{LB}_{\mu }}}{m_{LB}}.$$

The specific absorbed fraction for this case is

(29) $$\Phi \;(LB\leftarrow LB)=\frac{\phi \;(LB\;\leftarrow \;LB)}{m_{LB}}.$$

### 2.12. S-value calculations

This study follows the formalism of the MIRD schema (Bolch et al 2009). Within this system, for a specific radionuclide, the absorbed dose to a target region $r_{T}$ can be expressed as

(30) $$D\left(r_{T}\right)=\sum_{r_{S}}\tilde{A}\left(r_{S}\right)\;S\;\left(r_{T}\leftarrow r_{S}\right),$$

where $\tilde{A}\left(r_{S}\right)$ is the time-integrated activity (i.e. the total number of decays) within source region $r_{S}$, and $S\left(r_{T}\leftarrow r_{S}\right)$ is the S-value, representing the mean absorbed dose to the target region per nuclear decay in the source region. S-values are expressed as

(31) $$S\;\left(r_{T}\leftarrow r_{S}\right)=\sum_{i}E_{i}Y_{i}\Phi \;\left(r_{T}\leftarrow r_{S},E_{i}\right)=\sum_{i}E_{i}Y_{i}\frac{\phi \;\left(r_{T}\;\leftarrow \;r_{S},\;E_{i}\right)}{m_{r_{T}}},$$

where $E_{i}$ is the energy of the i th nuclear transformation of the radionuclide, $Y_{i}$ is the yield of this transformation, and $\Phi \;\left(r_{T}\leftarrow r_{S},E_{i}\right)$ and $\phi \;\left(r_{T}\leftarrow r_{S},E_{i}\right)$ are the specific absorbed fraction and absorbed fraction, respectively, for the source particle with energy $E_{i}$.

To calculate S-values, specific absorbed fractions for the four source-target combinations (LB←LB,LB←LP,LP←LB, and LP←LP) were first interpolated (with respect to source particle energy) via piecewise cubic Hermite interpolating polynomials (Fritsch and Carlson 1980). These resampled quantities were then spectrum-weighted and summed for each of the 22 therapeutic and 11 diagnostic radionuclides of interest. Radionuclide decay data (energies and yields) were taken from those published in the MIRD Monograph on Radionuclide Data and Decay Schemes (Eckerman and Endo 2007). For each radionuclide, radiation-specific S-values were computed for photons, betas, electrons, alphas, and alpha recoil particles, and summed for the overall S-value.

## 3. Results

### 3.1. Integration of macroscale vascular models into MRCPs

**Figure 7.**
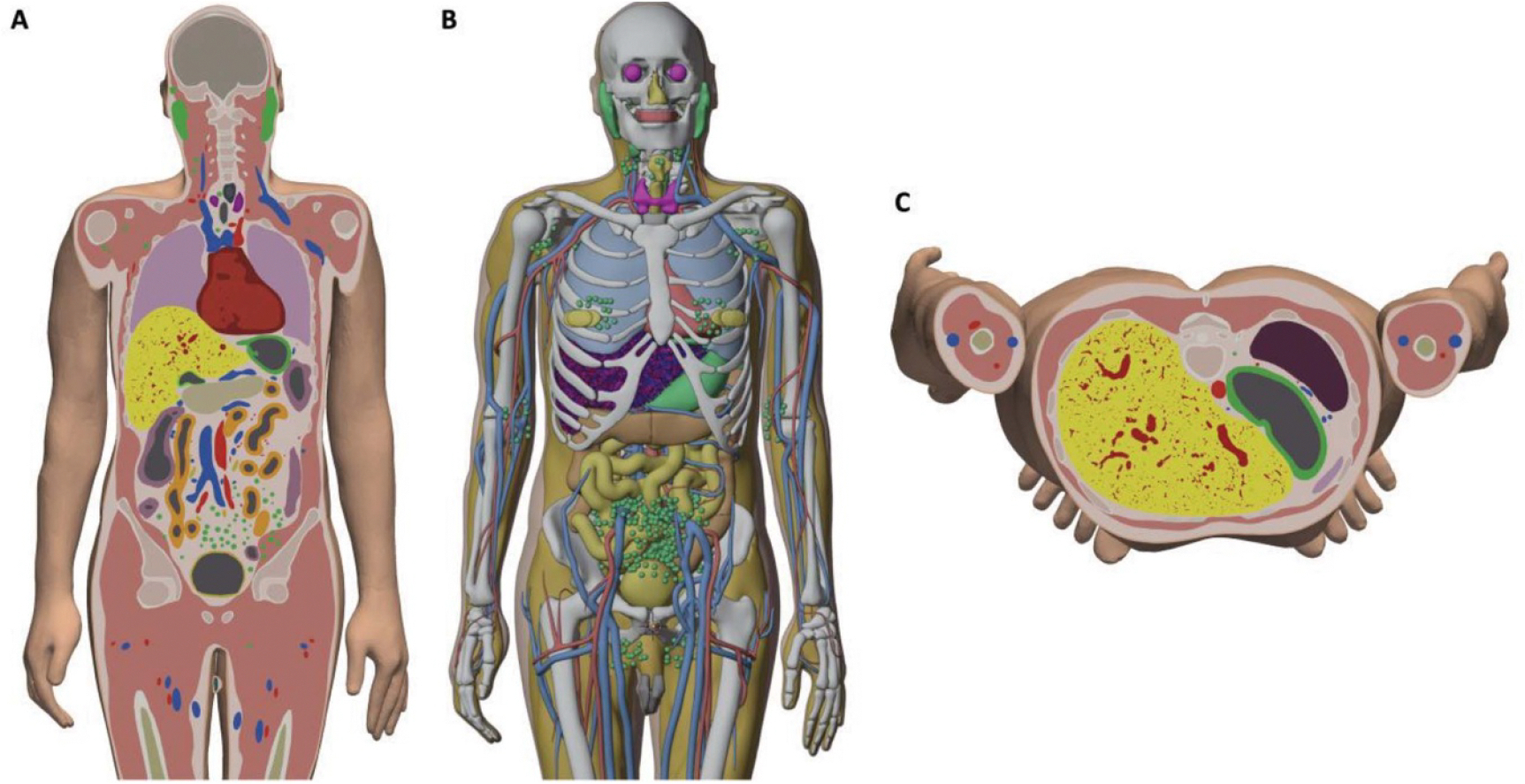
Polygon and tetrahedral mesh adult male MRCP with highly detailed liver vasculature. (A) Clipped coronal view of tetrahedralized whole-body phantom. (B) Perspective view of phantom with liver surface hidden to reveal organ vasculature. (C) Clipped axial view of tetrahedralized whole-body phantom.

**Figure 8.**
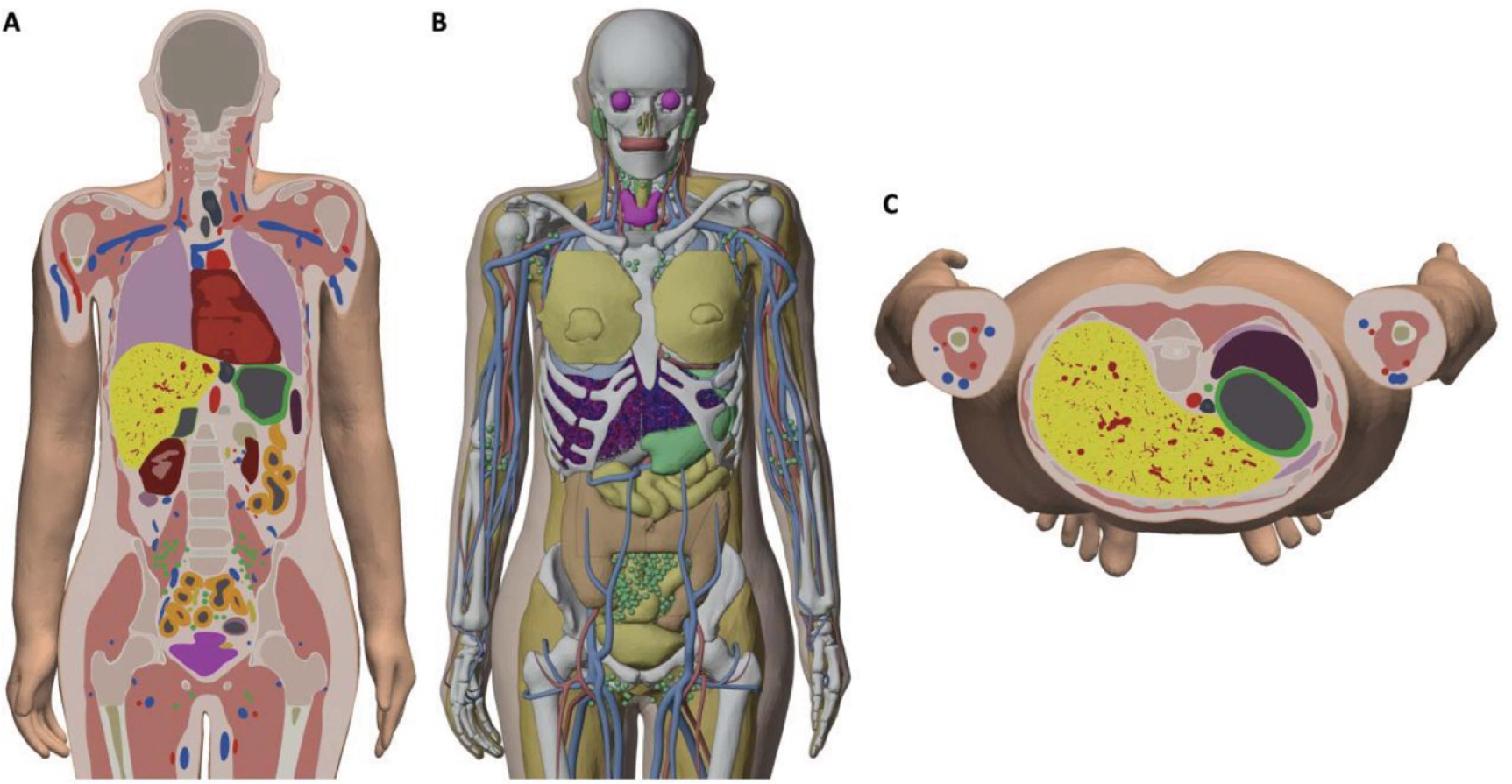
Polygon and tetrahedral mesh adult female MRCP with highly detailed liver vasculature. (A) Clipped coronal view of tetrahedralized whole-body phantom. (B) Perspective view of phantom with liver surface hidden to reveal organ vasculature. (C) Clipped axial view of tetrahedralized whole-body phantom.

The macroscale vascular tree models, in polygonal surface format, were installed into and tetrahedralized along with the adult MRCPs. Visualizations of the polygon and tetrahedral mesh whole-body phantoms can be seen in figures 7 and 8. The outer surface of the liver in each model was not altered from the liver surface present in the adult MRCPs.

### 3.2. Absorbed fractions and specific absorbed fractions

**Figure 9.**
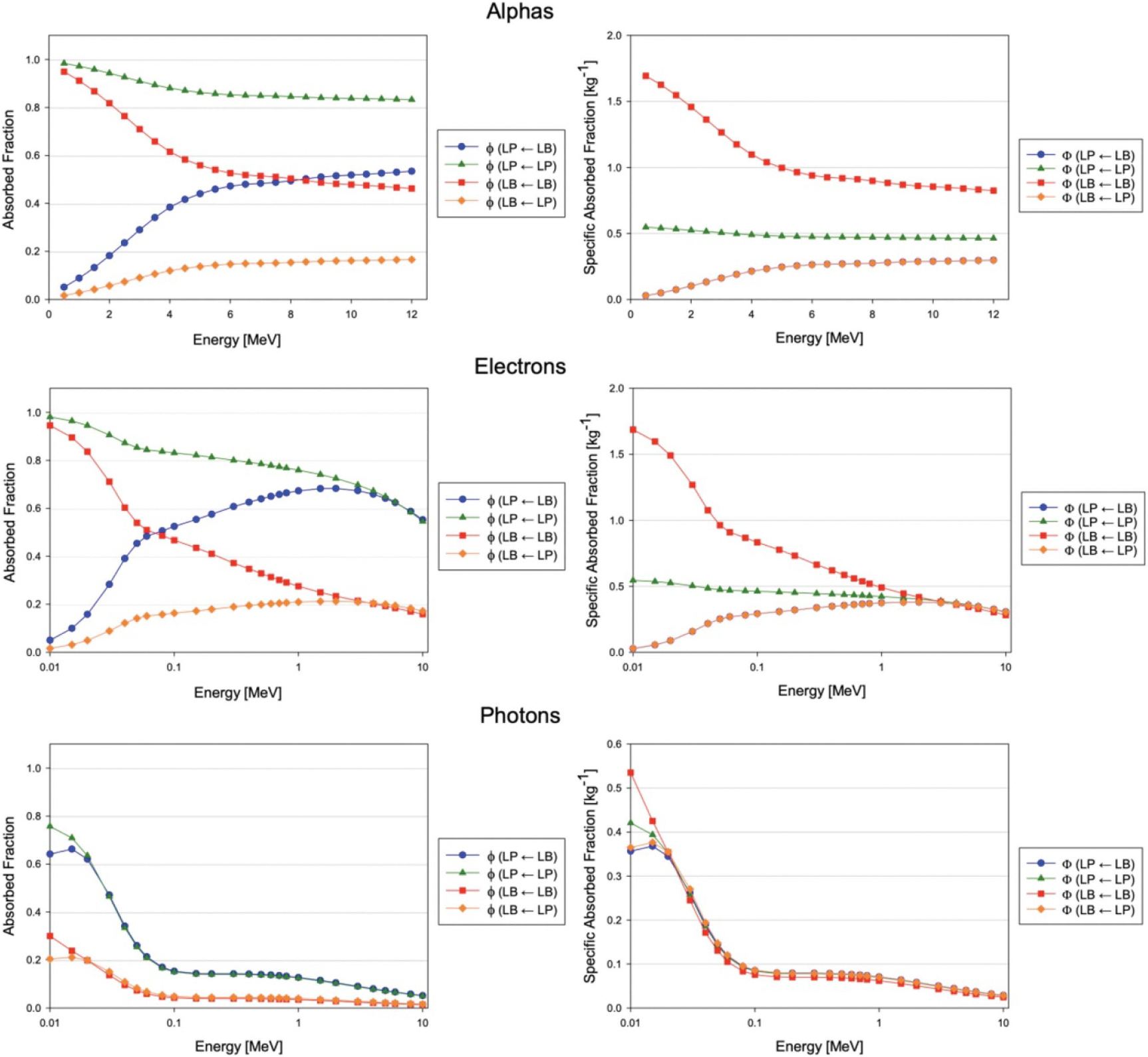
Absorbed fractions (left) and specific absorbed fractions (right) for blood and parenchyma sources of monoenergetic alphas, electrons, and photons within the adult male liver vasculature phantoms.

**Figure 10.**
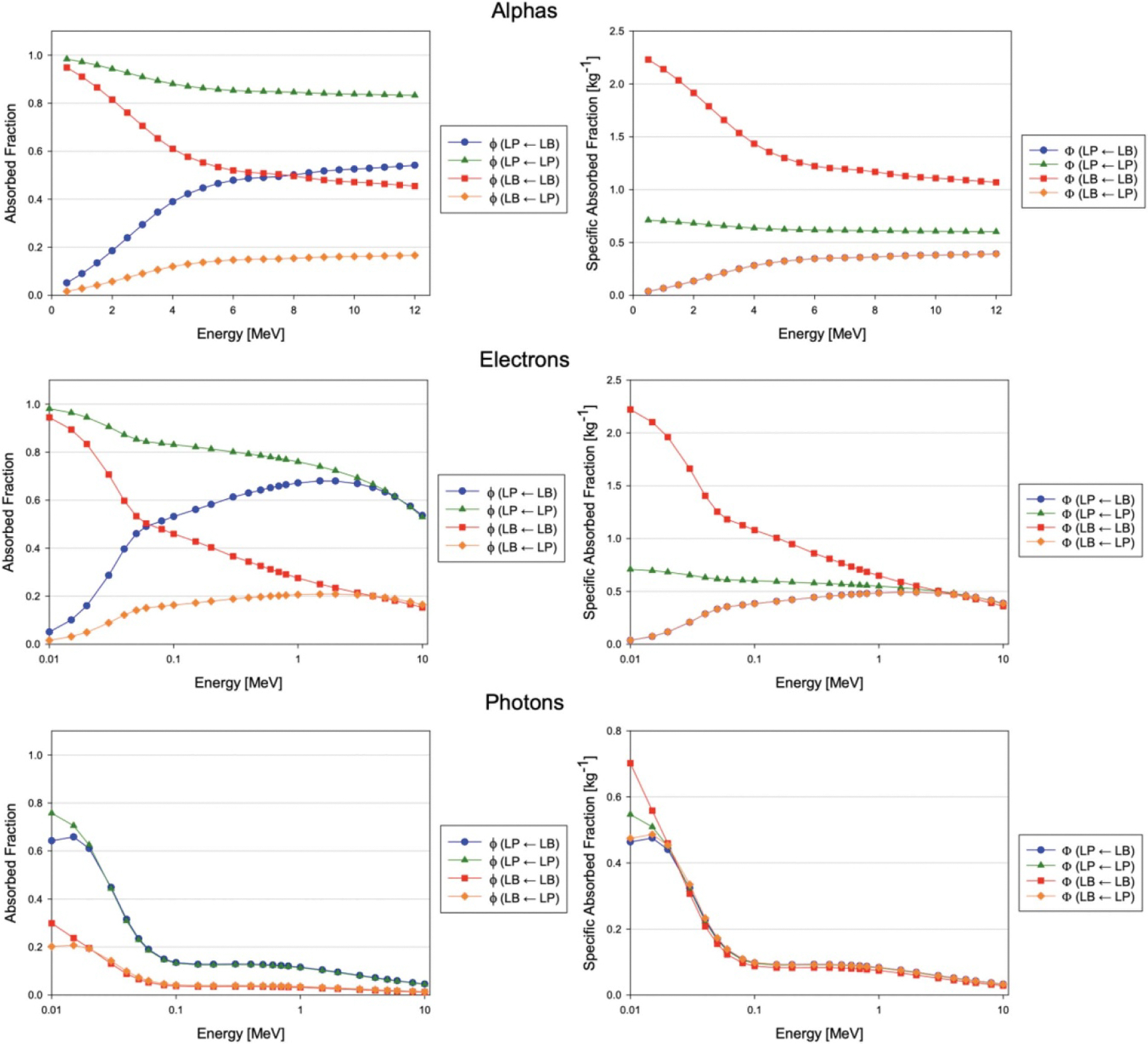
Absorbed fractions (left) and specific absorbed fractions (right) for blood and parenchyma sources of monoenergetic alphas, electrons, and photons within the adult female liver vasculature phantoms.

Absorbed fractions for blood and parenchyma sources and targets within the macroscale and microscale vascular models were computed using PHITS. These results were mathematically combined and used to calculate specific absorbed fractions (figures 9 and 10) using the methodology described previously. Note that in the plots of the specific absorbed fraction, it may appear that the Φ(LP←LB) plot is hidden; this is due to the mathematical equivalency of Φ(LP←LB) and Φ(LB←LP) as described by the reciprocity theorem and will be discussed later.

In the microscale model simulations, it was observed that at photon energies over 150 keV, the absorbed fraction to any tissue component (from any source) was approximately equal to the fractional volume occupied by that component. This was expected, as it is a well-known phenomenon which has been previously observed (Akabani et al 1991). Thus, simulations with photon sources were only performed for monoenergetic photons up to and including those with initial energies of 150 keV, and absorbed fractions for energies above this threshold were assumed to be equal to the respective volume fractions.

### 3.3. S-values for clinically relevant radionuclides

S-values were calculated using the specific absorbed fractions derived from the proposed multiscale Monte Carlo approach. S-values for the adult male for therapeutic and diagnostic radionuclides (see table 5) can be found in tables 6 and 7, respectively, while those for the adult female can be found in tables 8 and 9. To enable mass and relative biological effectiveness scaling, the full dataset, containing S-value contributions for each radiation type (alphas, betas, gammas), can be found in the electronic annex.

## 4. Discussion

### 4.1. Vessel morphologies and dosimetric implications

Similar trends for absorbed fractions and specific absorbed fractions were observed in the male and female coupled models for all particles. For electrons and photons, at high energies, absorbed fractions tended to decrease due to escape from the macroscale (whole liver) organ models. This coincided with the convergence of ϕ(LP←LB)≈ϕ(LP←LP) and ϕ(LB←LB)≈ϕ(LB←LP) as the absorbed fractions in the microscale model approached volume fractions as discussed previously, and is likely related to the particle ranges becoming large compared to the vessel diameters in both the micro- and macroscale models.

### 4.2. Generalizability of dosimetric coupling approach

The methodology presented in this work combines dosimetry at multiple scales to produce results which are spatial scale-independent. For example, the S-value computed for a radionuclide with the liver blood as source and liver parenchyma as target would represent the global average of absorbed dose in the parenchyma resulting from a radionuclide decay in the blood; the novelty of this approach is that this decay could have occurred in any blood vessel in the liver, regardless of size and including capillaries.

In the liver, the repetitive and pervasive structure of parenchymal microstructure provided a convenient environment in which only macroscale and microscale models needed to be constructed. The minimum radius for a vessel in the macroscale model was of the same order of magnitude of the maximum radius for a vessel in the microscale model, and thus, vessels of all sizes (and therefore all blood) within the liver were effectively modeled, precluding the need for an intermediate mesoscale vascular model. However, in organs which have a larger diversity of vessel and capillary diameters, a mesoscale model may be useful and can be introduced into this approach in a similar manner; a microscale model, with internal reflection boundaries, could be assumed to fill the parenchyma region in a mesoscale blood vessel model. The mesoscale model itself could then be simulated using reflective boundaries and assumed to fill the parenchyma region of a macroscale model (which allows for particle escape from the organ).

Many of the arguments which combine dosimetric contributions from the two different spatial scales should only be adopted with due consideration if applying to other organs. For example, the presence of air regions throughout the lungs would cause inaccuracies in many of the steps involving relative mass scaling of absorbed fractions. In cases such as these, the mass fraction scaling terms could be modified to include scaling by ratios of the typical ranges of the source particles in the respective tissues (e.g. air and parenchyma).

### 4.3. Validation of reciprocity theorem

Interestingly, the specific absorbed fractions for the two crossfire cases, Φ(LP←LB) and Φ(LB←LP), were essentially identical for all source particles at all energies, as seen in figures 8 and 9. This is a demonstration of the reciprocity theorem, which states that under certain conditions (generally related to lack of scattered radiations or uniformity of the medium), specific absorbed fractions between source and target regions are independent of which region is the source and which is the target (Cristy 1983, Petoussi-Henss et al 2007, Wayson et al 2012, Zankl et al 2012, Hertel and Jokisch 2019). Accordingly, since S-values depend on specific absorbed fractions, the S-values for the crossfire cases, S(LP←LB) and S(LB←LP), were approximately equal for all radionuclides examined.

The validity of the reciprocity theorem is likely to hold (approximately) for models comprised primarily of soft tissue and blood regions. Importantly, in future studies, this may reduce the number of Monte Carlo radiation transport simulations required to compute specific absorbed fractions for an entire blood-parenchyma system. For example, the dataset for one source-target combination (e.g. Φ(LP←LB)) can be computed via simulation; by invoking the reciprocity theorem, the values for the reverse case, Φ(LB←LP), are then known.

### 4.4. Absorbed fractions for a whole liver target

In general, the blood vessels within the macroscale models were homogeneously distributed throughout the liver. Analogously, the parenchyma (and therefore nested microscale structure) was also homogeneously distributed. It then follows that

(32) ϕ(LB←LB)+ϕ(LP←LB)≈ϕ(LB←LP)+ϕ(LP←LP),

which is equivalent to the statement

**Figure 11.**
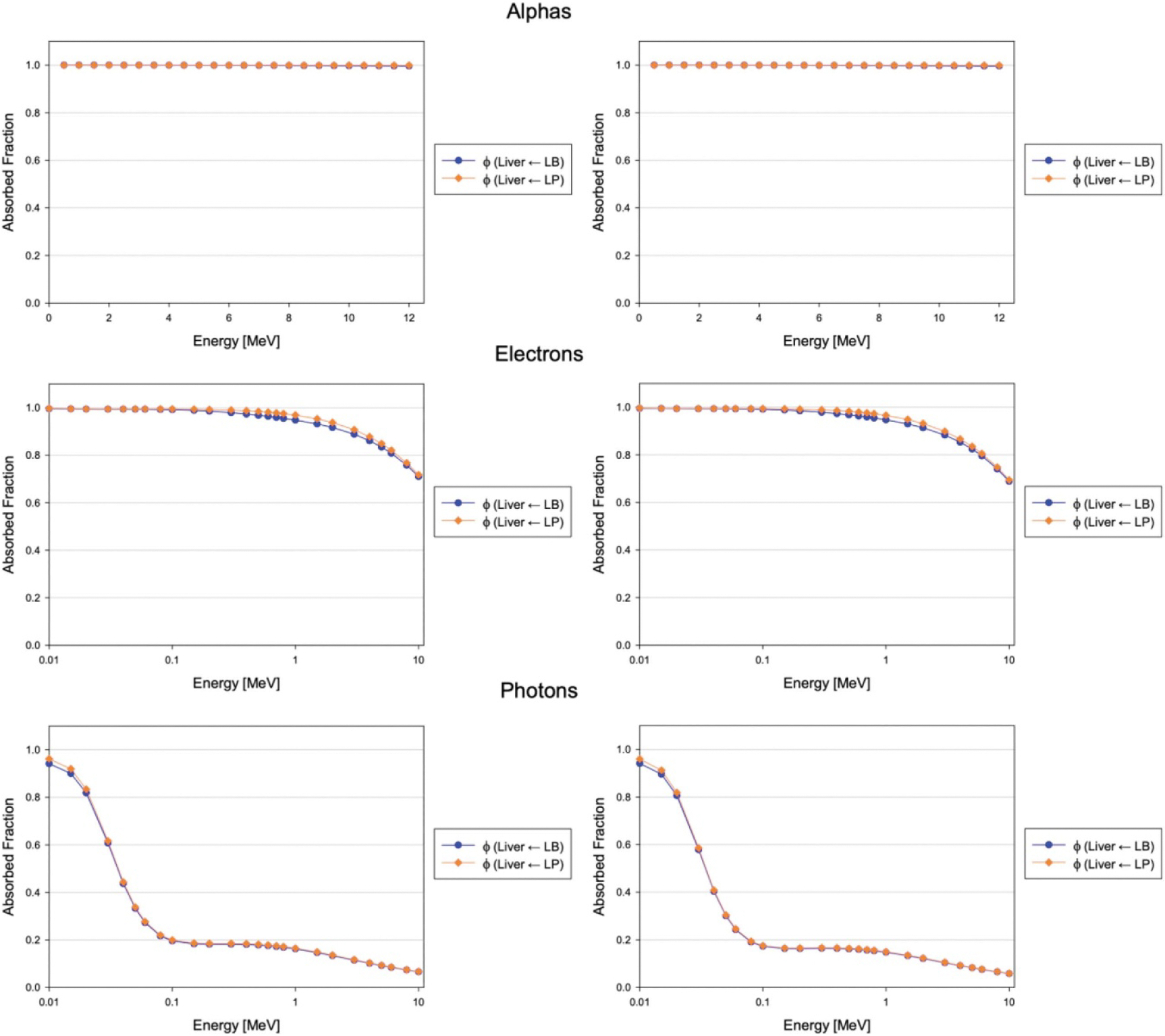
Absorbed fractions to the whole liver for blood and parenchyma sources of monoenergetic alphas, electrons, and photons within the adult male (left column) and adult female (right column) liver vasculature phantoms.

(33) ϕ(L←LB)≈ϕ(L←LP),

where L represents the entire liver. For low-energy source particles, these approximations should be very accurate since there is negligible escape from the liver. For higher energy particles, the expressions on each side of these approximations should begin to differ due to the slight heterogeneity of blood distribution throughout the liver; for example, since the major blood vessels entering and exiting the organ occur at the organ surface, it is expected that there is greater particle escape (and therefore lower absorbed fractions) with liver blood as the source. Indeed, the expected trends were validated and can be seen in figure 11.

### 4.5. Comparison to macroscale-only dosimetry

In a previous study, Correa et al (2022) developed macroscale intra-liver vasculature models for the adult male and female, also using the MRCPs. In these models, the interior of the liver was partitioned into two regions: liver inside blood vessels, LIBV, and liver outside blood vessels, LOBV. These regions are analogous to ${LB}_{M}$ and ${LCT}_{M}$, respectively, presented in the current study. Specific absorbed fractions from parenchyma to parenchyma and blood to parenchyma were then approximated as

(34) $$\Phi \;(LP\leftarrow LP)\approx \Phi \;(LOBV\leftarrow LOBV)=\frac{\phi \;(LOBV\;\leftarrow \;LOBV)}{m_{LOBV}}$$

and

(35) $$\Phi \;(LP\leftarrow LB)\approx \Phi \;(LOBV\leftarrow LB)=\frac{f_{BV}\;\cdot \;\phi \;(LOBV\;\leftarrow \;LIBV)\;+\;\left(1\;-\;f_{BV}\right)\;\cdot \;\phi \;(LOBV\;\leftarrow \;LOBV)}{m_{LOBV}},$$

where $f_{BV}$ is the mass fraction of total liver blood contained within the explicitly modeled macroscale vessels. In the notation of the present study, these equations become

(36) $$\Phi \;(LP\leftarrow LP)\approx \Phi \;\left({LCT}_{M}\leftarrow {LCT}_{M}\right)=\frac{\phi \;\left({LCT}_{M}\;\leftarrow \;{LCT}_{M}\right)}{m_{{LCT}_{M}}}$$

and

(37) $$\Phi \;(LP\leftarrow LB)\approx \Phi \left({LCT}_{M}\leftarrow LB\right)=\frac{f_{BV}\;\cdot \;\phi \;\left({LCT}_{M}\;\leftarrow \;{LB}_{M}\right)\;+\;\left(1\;-\;f_{BV}\right)\;\cdot \;\phi \;\left({LCT}_{M}\;\leftarrow \;{LCT}_{M}\right)}{m_{{LCT}_{M}}}.$$

**Figure 12.**
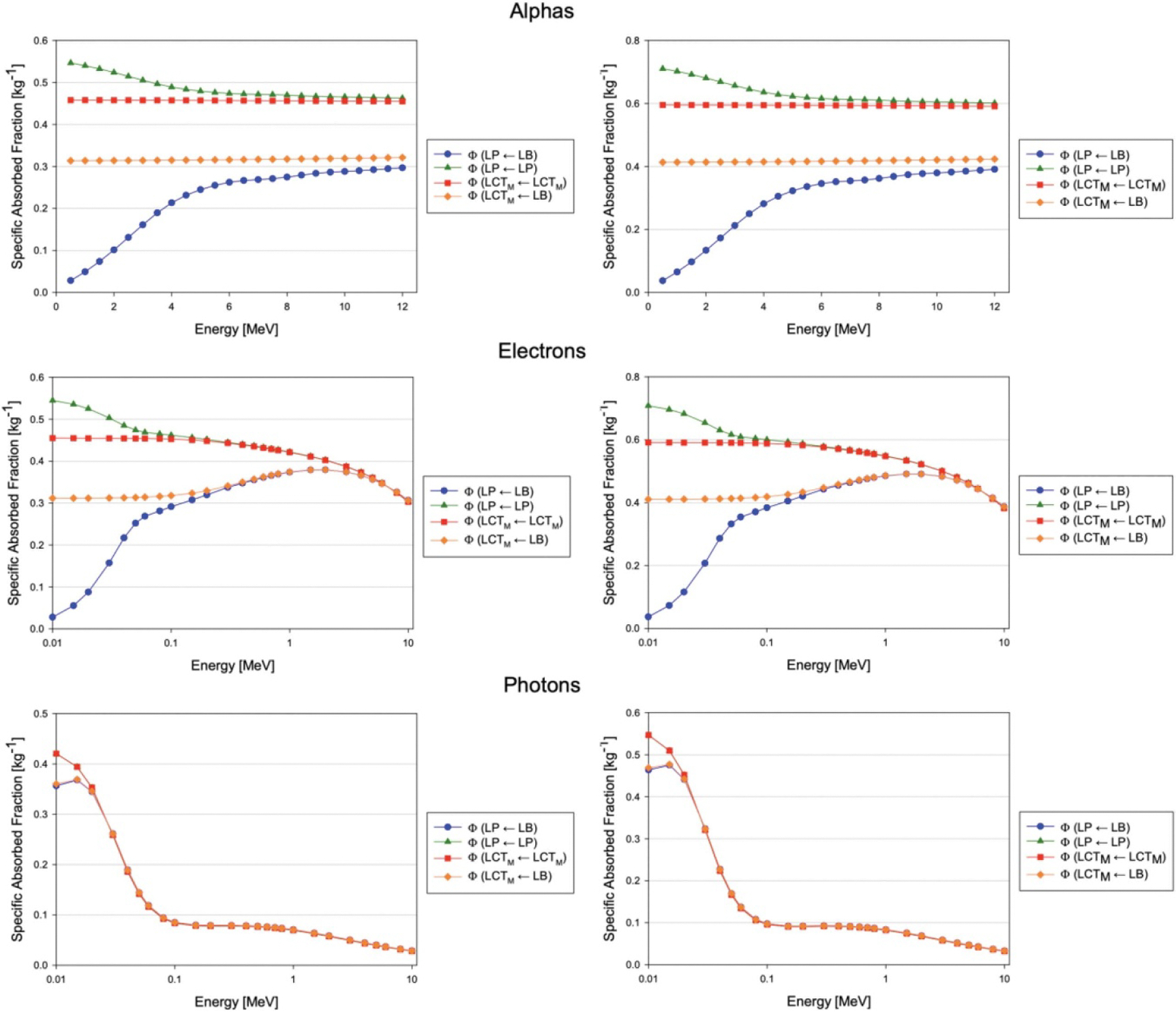
Specific absorbed fractions for the adult male (left column) and adult female (right column) assuming a multiscale or macroscale-only dosimetry model.

The approximations for Φ(LP←LP) and Φ(LP←LB) using this single-scale approach and the current multi-scale approach were compared (figure 12).

While the trends for photons were similar, there were marked differences in the specific absorbed fractions for electrons and alphas. These differences are significant for most alpha energies and for electrons under 100 keV, where the ranges of these particles become comparable to the diameters of blood vessels in the hepatic lobule. This strongly implies the importance and utility of incorporating a microscale vasculature model when assessing dose to blood and parenchyma in highly vascularized organs.

### 4.6. Limitations

The primary limitation of the microscale model is its stylized nature; all microscale structures are represented with the same repeating spatial orientation and dimensions. In reality, these tissues may exhibit significant variability in size, shape, and composition, and will possess randomness in their structure. Additionally, there is evidence for sex-based differences in liver vascularization and microstructure, such as a higher proportion of Kupffer cells in the female liver and larger, but relatively fewer hepatocytes in the male liver; these differences are likely due to hormonal differences and may further lead to microvascular remodeling (Marcos et al 2015, Ullah et al 2021, Goldfarb et al 2022a, Goldfarb et al 2022b, Smiriglia et al 2023). For simplicity, a single, sex-independent microscale model was used in this study. This approach was deemed reasonable given that the blood mass fractions in adult male and female livers are approximately equivalent, supporting its use in dosimetry studies. This model also lacks some other cell types and tissues, such as lymphatic vessels, which are either not sensitive to radiation, have generally unknown activity concentrations, or are microscopic and outside the capabilities of the physics macroscale cross-section libraries used by PHITS.

Future improvements to the microscale model may include informing it with spatial information coming from imaging or histological studies, though this presents a new set of challenges in constructing a tetrahedral mesh model that is usable in Monte Carlo radiation transport simulations.

The macroscale models are anatomically accurate in terms of vessel sizes and branching patterns. However, due to computational considerations, the walls of these vessels were not modeled. While this is acceptable for photons and electrons, short-range particles such as alphas may have slightly different dosimetric consequences; tallying energy deposition to vessel walls is likely to slightly reduce the absorbed fractions to the parenchyma. Modeling vessel walls using the described approach is feasible and would involve a vertex normal-oriented offset operation across all facets of the vasculature mesh by a prescribed thickness as a function of vessel radius (with potential corrections for induced mesh intersections); this would effectively double the size of the model in terms of storage and RAM demands in applications such as Monte Carlo simulations.

It is important to note that the models presented in this study represent hepatic tissues specifically in healthy adults. However, these methods could easily be applied to pediatric individuals, potentially based on the recently developed pediatric MRCPs (Choi et al 2021). Furthermore, patient- or population-specific information regarding tissue composition could be used to refine the material definitions in the phantoms and simulations used to generate dosimetric quantities such as S-values. For example, fatty liver disease, which has a global prevalence of around 32% among adults, could be modeled by appropriately weighting the adipose material term for parenchyma in Monte Carlo simulations (Teng et al 2023). Also, while hepatocytes were defined as a ‘fill’ region outside of the other explicitly modeled structures in the microscale model, the stromal (inter-hepatocyte) region comprised of endothelial and hepatic stellate cells, among others, could be explicitly modeled in the future to further partition the LP region and better quantify microscopic dose heterogeneity (Heindryckx and Gerwins 2015).

### 4.7. Coupled model applications—radiopharmaceutical dosimetry

This approach to microscale-macroscale dosimetry coupling with respect to organ vasculature has significant applications to radiopharmaceutical therapy and potentially also dose assessment in diagnostic nuclear medicine. Dose assessment following non-medical exposures to radionuclides (e.g. accidental inhalation) could also be enhanced by integrating information from multiple spatial scales. In effect, this approach allows the MIRD schema to be extended to quantitatively distinguish dose contributions to and from blood and parenchyma within vascularized organs, assuming a set of multiscale models which collectively account for all blood within the organ and quantified activity in each subregion. Multiscale vascular models for various organs could then inform radiopharmaceutical dosimetry platforms such as the publicly available software MIRDcalc (Carter et al 2023, Kesner et al 2023). Reliable quantitative dosimetry estimates would be predicated on the ability to quantitatively assess activity concentration in regions of blood and parenchyma to obtain their associated values for time-integrated activity.

Contemporary nuclear medicine dosimetry workflow is already readily amenable to incorporating the coupled microscale-macroscale model. The activity concentration in blood is routinely used in RPT dosimetry as an indicator of the activity concentration in the red marrow (which is often dose-limiting), and may be obtained via, e.g. serial blood draws or quantitative tomographic nuclear medicine imaging with volumes-of-interest (VOI) placed over the cardiac contents. The volume and mean activity concentration associated with whole organs (inclusive of blood contained within the organs) is typically also evaluated from quantitative imaging VOI analysis. Therefore, given the known blood activity concentration C(Blood), the known blood-inclusive organ activity concentration C(Organ), known blood inclusive organ volume V(Organ), and assuming reference volume-fractional blood content for the organ $f_{\text{Blood}}$ (Organ), the activity associated with the blood component of the organ is the following, using the present example of the liver for illustrative purposes:

(38) $$A\;(LB)=C\;(\text{Blood})\;\cdot \;V(L)\;\cdot \;f_{\text{Blood}}\;(L).$$

Then, the activity associated with the parenchyma component of the liver follows as:

(39) A(LP)=C(L)⋅V(L)-A(LB).

The absorbed dose rate for the liver parenchyma target then follows as:

(40) $$\dot{D}\;(LP)=\left[\sum_{r_{S}⊈L}A\;\left(r_{S}\right)\;S\;\left(L\leftarrow r_{S}\right)\right]+A\;(LP)\;S\;(LP\leftarrow LP)+A\;(LB)\;S\;(LP\leftarrow LB),$$

where, note, the liver self-dose term in the summation has been omitted and replaced with the separate contributions of liver blood and liver parenchyma sources; the required S-values may be obtained from tables 6–9.

### 4.8. Coupled model applications—Y-90 microsphere radioembolization

This coupled liver model could be used to simulate Y-90 radioembolization treatments. In this procedure, Y-90 labeled microspheres are injected into the hepatic artery with a twofold aim: to cut off the tumor nutrients supply by embolizing the tumor-feeding vasculature, and to kill the tumor cells by emitting radiation (Gulec and McGoron 2022). Previous studies have noted the difficulty in reliably predicting organ-level function (or loss thereof) using commonly used clinical metrics following these treatments, with hepatobiliary scintigraphy proposed as a method to determine local differences in microsphere activity (Braat et al 2017). Thus, a multiscale, multicompartment model as the one presented here can be paired with such imaging modalities to better estimate local distributions of absorbed dose in the liver.

The role of macroscale models of the liver vasculature has been recently investigated to simulate the pathway of microspheres through the liver vasculature using a Markov chain Monte Carlo approach (Huesa-Berral et al 2024). It allows predicting the amount of activity delivered (number of microspheres) in the tumor and normal tissue. In this line, the macroscale model can inform the microscale model about the activity delivered, and the microscale model can be used to locally analyze the spatial distribution of microspheres in the hepatic lobe and its impact on the dose distribution.

### 4.9. Coupled model applications—lymphocyte dosimetry in external beam radiotherapy

The macroscale vasculature model could also be applied in the context of blood flow tracking through the organ, for example to compute blood dose in external beam radiotherapy of liver cancer patients (Xing et al 2022). This has been done in the context to explain radiation-induced lymphopenia (McCullum et al 2023). However, it is unclear to what extent blood dose is representative of lymphocyte dose (Beekman et al 2023), as the flux of lymphocytes through the microvasculature may be impeded compared to blood flow due to their size in relation to the diameter of the liver sinusoids and potential molecular interactions with the endothelial cells (Downey et al 1990, Hogg and Doerschuk 1995, Kuebler and Goetz 2002). The microscale model could also provide a mesh-based environment to study these mechanisms using computational fluid dynamics (Salman and Yalcin 2021, He et al 2022, Kamada et al 2022).

To perform blood particle tracking as for the case of circulating lymphocytes, the underlying centerline graph of the blood vessel must be used and must form a closed system, such that a single particle can enter the network, circulate for some time, and then exit the network. For this reason, an equal number of total endpoints were generated for each of the three vascular trees, and point triplets were computed with one endpoint from each of the three trees. This procedure involved determining a one-to-one mapping of points in 3D space from the arterial to venous systems and from the portal venous to venous systems. This task represents a form of the assignment problem, and the optimal mappings were determined by implementing in Python a modified form of the Hungarian algorithm (Kuhn 1955). Though not explicitly utilized in this study, the closed centerline graphs for these liver vasculature models, as well as the computational framework for creating such graphs, are available for future studies related to explicit 4D blood particle tracking.

## 5. Conclusions

This study presents a novel computational framework for coupling radiation transport calculations across multiple spatial scales in vascularized organs, with specific application to liver dosimetry. The developed approach represents a significant advancement over current homogeneous organ models by explicitly accounting for both blood and parenchymal tissue distributions across macro- and microscale domains. The mathematical framework successfully combines absorbed fractions from independent Monte Carlo simulations at different spatial scales, enabling the calculation of blood-specific and parenchyma-specific S-values for therapeutic and diagnostic radionuclides.

The validation of the reciprocity theorem for crossfire between blood and parenchyma suggests potential computational efficiency gains in future applications. The demonstrated framework’s ability to account for 100% of organ blood content while maintaining anatomical accuracy makes it particularly valuable for applications in radiopharmaceutical therapy dosimetry, where the spatial distribution of activity between blood and parenchyma significantly impacts dose calculations.

The coupled model system has immediate applications in improving dose calculations for Y-90 microsphere radioembolization treatments and analyzing blood dose during external beam radiotherapy. While developed specifically for the liver, the mathematical framework presented here can be adapted for other vascularized organs, potentially with the addition of mesoscale models where anatomically appropriate. Future work may focus on incorporating more detailed microscale structures based on imaging or histological data.

This work represents an important step toward more accurate organ-specific dosimetry that accounts for tissue heterogeneity at multiple spatial scales. The developed models and methods provide a foundation for improved dose-effect relationships in both diagnostic and therapeutic nuclear medicine applications, potentially leading to more effective treatment planning and delivery.

## Figures and Tables

**Table 1.** Masses for the various regions in the combined liver vasculature models. Nominal values represent those derived from ICRP Publication 145.

	Mass (g)		
	Male	Female	Symbol

Liver (nominal)	2360.00	1810.00	
Liver blood (nominal)	560.00	410.00	
Liver parenchyma (nominal)	1800.00	1400.00	
Liver (actual)	2360.00	1809.99	$m_{L}$
Liver blood (actual)	560.48	425.26	$m_{\mathrm{LB}}$
Liver parenchyma (actual)	1799.52	1384.73	$m_{\mathrm{LP}}$
Liver blood (macroscale)	176.70	129.96	$m_{{\mathrm{LB}}_{M}}$
Liver composite tissue (macroscale)^a^	2183.28	1679.99	$m_{{\mathrm{LCT}}_{M}}$
Liver blood (microscale)	383.77	295.31	$m_{{\mathrm{LB}}_{\mu }}$

a Inclusive of microscale blood.

**Table 2.** Masses and mass fractions for the tissue components within the microscale liver lobule model. This distribution was assumed to be uniform throughout the liver parenchyma outside of the macroscopic blood vessels.

Microscale tissue component	Mass (g)	Mass fraction

Central vein	1.23 × 10^−06^	1.50%
Portal arteries	5.04 × 10^−08^	0.06%
Portal veins	4.50 × 10^−07^	0.55%
Bile ducts	7.12 × 10^−08^	0.09%
Bile canaliculi	4.36 × 10^−06^	5.30%
Sinusoids	1.26 × 10^−05^	15.38%
Space of Disse	2.77 × 10^−06^	3.37%
Kupffer cells	1.05 × 10^−07^	0.13%
Hepatocytes	6.05 × 10^−05^	73.63%
Total	8.22 × 10^−05^	100.00%

**Table 3.** Elemental mass fractions and densities for the tissue components within the macroscale and microscale liver vasculature models.

		Elemental mass fraction (%)										Density (g cm^−3^)
		H	C	N	O	Na	P	S	Cl	K	Fe	

*Macroscale components*	ICRP 145 liver	10.20	13.20	3.10	72.30	0.20	0.20	0.30	0.20	0.30	0.00	1.060
	ICRP 145 blood	10.20	11.00	3.30	74.50	0.10	0.10	0.20	0.30	0.20	0.10	1.060
	Parenchyma (derived)	10.20	13.38	3.08	72.12	0.21	0.21	0.31	0.19	0.31	0.00	1.060

*Microscale components*	Central vein	10.20	11.00	3.30	74.50	0.10	0.10	0.20	0.30	0.20	0.10	1.060
	Portal arteries	10.20	11.00	3.30	74.50	0.10	0.10	0.20	0.30	0.20	0.10	1.060
	Portal veins	10.20	11.00	3.30	74.50	0.10	0.10	0.20	0.30	0.20	0.10	1.060
	Bile ducts	10.80	4.10	1.10	83.20	0.30	0.00	0.10	0.40	0.00	0.00	1.000
	Bile canaliculi	10.20	11.00	3.30	74.50	0.10	0.10	0.20	0.30	0.20	0.10	1.000
	Sinusoids	10.80	4.10	1.10	83.20	0.30	0.00	0.10	0.40	0.00	0.00	1.060
	Space of Disse	10.80	4.10	1.10	83.20	0.30	0.00	0.10	0.40	0.00	0.00	1.000
	Kupffer cells	10.14	14.42	3.25	70.92	0.21	0.24	0.34	0.16	0.34	0.00	1.068
	Hepatocytes	10.14	14.42	3.25	70.92	0.21	0.24	0.34	0.16	0.34	0.00	1.068
	Total	10.20	13.38	3.08	72.12	0.21	0.21	0.31	0.19	0.31	0.00	1.060

**Table 4.** Details regarding Monte Carlo radiation transport simulations for both the macroscale and microscale liver vasculature models.

Item	Description	References

*Code and version*	PHITS v3.24	Sato et al (2018, 2023)
*Source description*	*Macroscale*: Tetrahedral source regions (s-type = 24). Particles were produced uniformly from each tetrahedron, which belong to the specified universe.	Sato et al (2018, 2023)
	*Microscale*: Random points within a barycentric coordinate system were generated for each tissue region and used as dump file sources.	
*Cross sections*	EGS5 for photons, electrons, and positrons INCL for nucleons and light ions	Hirayama et al (2005)
*Transport parameters*	*Photons and electrons*: Primary and secondary particles tracked with an energy cutoff of 1 keV *Alphas*: Tracked with an energy cutoff of 1 keV/nucleon	Sato et al (2018, 2023)
*Variance reduction*	No variance reduction techniques were utilized in this study.	
*Statistical uncertainties and number of histories*	Histories for photons, electrons, and alphas were set at 10^7^ primary source particles for all macroscale and microscale radiation transport simulations. Relative errors for all tallied quantities were less than 1%.	Sato et al (2018, 2023)
*Data and post processing*	Energy deposited (in MeV/source) was tallied in all tissue regions in both the macroscale and microscale simulations. At the macroscale, the liver was partitioned only into blood and parenchyma and thus no post-processing was required to compute absorbed fractions, aside from division of the energy deposited by the source particle energy. At the microscale, the nine tissue regions were grouped into blood and parenchyma source and target regions and appropriately mass- or volume-weighted to produce final absorbed fraction estimates.	Sato et al (2018, 2023)

**Table 5.** Therapeutic radionuclides considered in this study. For alpha emitters, continuous-slowing-down approximation (CSDA) ranges in water were taken from the NIST ASTAR database.^9^ For electron emitters, CSDA ranges in soft tissue (as defined in ICRU Report 46) were taken from the NIST ESTAR database (International Commission on Radiation Units and Measurements 1992).^10^

Radionuclide	Physical half-life	Principal therapy radiations	*R*_CSDA_ in tissue

Alpha-emitters			
211At	7.21 h	5.87 MeV *α* (211At)	48 *μ*m
		7.45 MeV *α* (211Po)	
212Bi	1.01 h	6.06 MeV *α* (212Bi)	50 *μ*m
		8.78 MeV *α* (212Po)	
213Bi	45.6 m	8.79 MeV *α* (213Bi)	91 *μ*m
		8.38 MeV *α* (213Po)	
223Ra	11.4 d	5.71 MeV *α* (223Ra)	46 *μ*m
		6.82 MeV *α* (219Rn)	
		7.39 MeV *α* (215Po)	
		6.62 MeV *α* (211Bi)	
225Ac	10.0 d	5.73 MeV *α* (225Ac)	47 *μ*m
		5.79 MeV *α* (221Fr)	46 *μ*m
		5.83 MeV *α* (225Ac)	48 *μ*m
		6.13 MeV *α* (221Fr)	51 *μ*m
		6.34 MeV *α* (217At)	54 *μ*m
		7.07 MeV *α* (217At)	64 *μ*m
		5.87 MeV *α* (213Bi)	48 *μ*m
		8.38 MeV *α* (213Po)	85 *μ*m
227Th	18.7 d	5.70 MeV *α* (227Th)	46 *μ*m
		5.71 MeV *α* (227Th)	46 *μ*m
		5.76 MeV *α* (227Th)	47 *μ*m
		5.98 MeV *α* (227Th)	49 *μ*m
		6.04 MeV *α* (227Th)	50 *μ*m
Beta-emitters			
89Sr	50.5 d	*β*− (E_max = 1490 keV)	7.1 mm
		(E_ave = 583 keV)	2.2 mm
90Y	2.67 d	*β*− (E_max = 2280 keV)	11 mm
		(E_ave = 934 keV)	4.0 mm
124I	4.18 d	*β*+ (E_max = 610 keV)	23 mm
		(E_ave = 188 keV)	0.41 mm
131I	8.02 d	*β*− (E_max = 610 keV)	23 mm
		(E_ave = 182 keV)	0.39 mm
153Sm	46.5 h	*β*− (E_max = 705 keV)	2.8 mm
		(E_ave = 225 keV)	0.54 mm
166Ho	26.8 h	*β*− (E_max = 1854 keV)	9.0 mm
		(E_ave = 672 keV)	2.0 mm
177Lu	6.65 d	*β*− (E_max = 500 keV)	1.8 mm
		(E_ave = 134 keV)	0.23 mm
186Re	3.72 d	*β*− (E_max = 1070 keV)	4.8 mm
		(E_ave = 323 keV)	0.94 mm
188Re	17.0 h	*β*− (E_max = 2120 keV)	10 mm
		(E_ave = 765 keV)	3.1 mm
Auger electron-emitters			
103Pd	17.0 d	AE (2–22 keV) (7.44 nt^−1^) from 103 Pd	0.02 *μ*m (0.26 keV AE)
		AE (16–40 keV) (0.99 nt^−1^) from 103 mRh	
111In	2.80 d	AE (40 eV–26 keV) (7.43 nt^−1^)	0.02 *μ*m (0.35 keV AE)
		CE (144–245 keV) (0.16 nt^−1^)	520 *μ*m (219 keV CE)
117 mSn	13.8 d	AE (10 eV–28 keV) (14.2 nt^−1^)	0.02 *μ*m (0.4 keV AE)
		CE (126–313 keV) (1.15 nt^−1^)	215 *μ*m (127 keV CE)
123I	13.3 h	AE (20 eV–30 keV) (13.7 nt^−1^)	0.02 *μ*m (0.45 keV AE)
		CE (127–1068 keV) (0.16 nt^−1^)	215 *μ*m (154 keV CE)
125I	59.4 d	AE (20 eV–30 keV) (23.0 nt^−1^)	0.02 *μ*m (0.45 keV AE)
		CE (3.7–36 keV) (0.945 nt^−1^)	18.2 *μ*m (30.6 keV CE)
193 mPt	4.33 d	AE (40 eV–74 keV) (27.4 nt^−1^)	0.016 *μ*m (70 eV AE)
		CE (1–135 keV) (2.99 nt^−1^)	2.5 *μ*m (10 keV CE)
195 mPt	4.02 d	AE (40 eV–74 keV) (36.6 nt^−1^)	0.015 *μ*m (60 eV AE)
		CE (5.9–239 keV) (2.78 nt^−1^)	8.8 *μ*m (20.3 keV CE)

**Table 6.** S-values for therapeutic radionuclides for the adult male.

Radionuclide	*S*-values (mGy/MBq-s)			
	*S* (LB ← LB)	*S* (LB ← LP)	*S* (LP ← LB)	*S* (LP ← LP)

*Alpha emitters*				
At-211	3.87 × 10^−04^	1.03 × 10^−04^	1.03 × 10^−04^	1.92 × 10^−04^
(Po-211)	1.13 × 10^−03^	3.23 × 10^−04^	3.23 × 10^−04^	5.75 × 10^−04^
(Bi-207)	2.75 × 10^−05^	2.46 × 10^−05^	2.46 × 10^−05^	2.61 × 10^−05^
Bi-212	3.82 × 10^−04^	1.22 × 10^−04^	1.23 × 10^−04^	2.04 × 10^−04^
(Po-212)	1.28 × 10^−03^	3.97 × 10^−04^	3.98 × 10^−04^	6.73 × 10^−04^
(T1-208)	8.08 × 10^−05^	6.68 × 10^−05^	6.70 × 10^−05^	7.30 × 10^−05^
Bi-213	6.09 × 10^−05^	3.17 × 10^−05^	3.17 × 10^−05^	4.15 × 10^−05^
(Po-213)	1.24 × 10^−03^	3.74 × 10^−04^	3.74 × 10^−04^	6.44 × 10^−04^
(T1-209)	7.99 × 10^−05^	6.32 × 10^−05^	6.33 × 10^−05^	7.01 × 10^−05^
(Pb-209)	2.21 × 10^−05^	1.04 × 10^−05^	1.04 × 10^−05^	1.42 × 10^−05^
Ra-223	9.10 × 10^−04^	2.39 × 10^−04^	2.39 × 10^−04^	4.49 × 10^−04^
(Rn-219)	1.03 × 10^−03^	2.91 × 10^−04^	2.91 × 10^−04^	5.23 × 10^−04^
(Po-215)	1.12 × 10^−03^	3.20 × 10^−04^	3.20 × 10^−04^	5.70 × 10^−04^
(Pb-211)	4.24 × 10^−05^	2.68 × 10^−05^	2.68 × 10^−05^	3.24 × 10^−05^
(Bi-211)	1.01 × 10^−03^	2.81 × 10^−04^	2.81 × 10^−04^	5.08 × 10^−04^
(T1-207)	4.46 × 10^−05^	2.86 × 10^−05^	2.86 × 10^−05^	3.44 × 10^−05^
(Po-211)	1.13 × 10^−03^	3.23 × 10^−04^	3.23 × 10^−04^	5.75 × 10^−04^
Ac-225	9.14 × 10^−04^	2.42 × 10^−04^	2.42 × 10^−04^	4.52 × 10^−04^
(Fr-221)	9.74 × 10^−04^	2.69 × 10^−04^	2.69 × 10^−04^	4.89 × 10^−04^
(At-217)	1.08 × 10^−03^	3.05 × 10^−04^	3.05 × 10^−04^	5.46 × 10^−04^
Th-227	9.35 × 10^−04^	2.50 × 10^−04^	2.50 × 10^−04^	4.64 × 10^−04^
*Beta and positron emitters*				
Sr-89	5.06 × 10^−05^	3.41 × 10^−05^	3.41 × 10^−05^	4.02 × 10^−05^
Y-90	7.24 × 10^−05^	5.57 × 10^−05^	5.58 × 10^−05^	6.27 × 10^−05^
I-124	2.83 × 10^−05^	2.45 × 10^−05^	2.45 × 10^−05^	2.63 × 10^−05^
I-131	2.62 × 10^−05^	1.48 × 10^−05^	1.49 × 10^−05^	1.86 × 10^−05^
Sm-153	3.33 × 10^−05^	1.49 × 10^−05^	1.49 × 10^−05^	2.09 × 10^−05^
Ho-166	6.13 × 10^−05^	4.09 × 10^−05^	4.10 × 10^−05^	4.84 × 10^−05^
Lu-177	1.88 × 10^−05^	7.82 × 10^−06^	7.82 × 10^−06^	1.13 × 10^−05^
Re-186	3.40 × 10^−05^	1.90 × 10^−05^	1.90 × 10^−05^	2.41 × 10^−05^
Re-188	6.50 × 10^−05^	4.68 × 10^−05^	4.69 × 10^−05^	5.39 × 10^−05^
*Auger electron emitters*				
Pd-103	2.37 × 10^−06^	8.42 × 10^−07^	8.19 × 10^−07^	1.32 × 10^−06^
In-111	1.05 × 10^−05^	7.39 × 10^−06^	7.37 × 10^−06^	8.48 × 10^−06^
Sn-117 m	2.38 × 10^−05^	1.02 × 10^−05^	1.02 × 10^−05^	1.46 × 10^−05^
I-123	7.32 × 10^−06^	4.09 × 10^−06^	4.06 × 10^−06^	5.13 × 10^−06^
I-125	6.68 × 10^−06^	2.14 × 10^−06^	2.08 × 10^−06^	3.53 × 10^−06^
Pt-193 m	2.15 × 10^−05^	5.92 × 10^−06^	5.92 × 10^−06^	1.08 × 10^−05^
Pt-195 m	3.25 × 10^−05^	8.30 × 10^−06^	8.28 × 10^−06^	1.59 × 10^−05^

**Table 7.** S-values for diagnostic radionuclides for the adult male.

Radionuclide	*S*-values (mGy/MBq-s)			
	*S* (LB ← LB)	*S* (LB ← LP)	*S* (LP ← LB)	*S* (LP ← LP)

*SPECT radionuclides*				
Ga-67	8.15 × 10^−06^	3.65 × 10^−06^	3.65 × 10^−06^	5.11 × 10^−06^
Tc-99m	4.02 × 10^−06^	2.34 × 10^−06^	2.33 × 10^−06^	2.90 × 10^−06^
In-111	1.05 × 10^−05^	7.39 × 10^−06^	7.37 × 10^−06^	8.48 × 10^−06^
I-123	7.32 × 10^−06^	4.09 × 10^−06^	4.06 × 10^−06^	5.13 × 10^−06^
*PET radionuclides*				
C-11	4.85 × 10^−05^	3.42 × 10^−05^	3.43 × 10^−05^	3.95 × 10^−05^
N-13	5.62 × 10^−05^	4.09 × 10^−05^	4.09 × 10^−05^	4.67 × 10^−05^
O-15	7.22 × 10^−05^	5.60 × 10^−05^	5.61 × 10^−05^	6.27 × 10^−05^
F-18	3.69 × 10^−05^	2.52 × 10^−05^	2.53 × 10^−05^	2.94 × 10^−05^
Ga-68	7.02 × 10^−05^	5.56 × 10^−05^	5.57 × 10^−05^	6.18 × 10^−05^
Rb-82	1.12 × 10^−04^	9.88 × 10^−05^	9.89 × 10^−05^	1.06 × 10^−04^

**Table 8.** S-values for therapeutic radionuclides for the adult female.

Radionuclide	*S*-values (mGy/MBq-s)			
	*S* (LB ← LB)	*S* (LB ← LP)	*S* (LP ← LB)	*S* (LP ← LP)

*Alpha emitters*				
At-211	5.04 × 10^−04^	1.36 × 10^−04^	1.36 × 10^−04^	2.49 × 10^−04^
(Po-211)	1.47 × 10^−03^	4.25 × 10^−04^	4.26 × 10^−04^	7.47 × 10^−04^
(Bi-207)	3.43 × 10^−05^	2.98 × 10^−05^	2.99 × 10^−05^	3.19 × 10^−05^
Bi-212	4.98 × 10^−04^	1.61 × 10^−04^	1.61 × 10^−04^	2.66 × 10^−04^
(Po-212)	1.66 × 10^−03^	5.24 × 10^−04^	5.24 × 10^−04^	8.75 × 10^−04^
(T1-208)	1.03 × 10^−04^	8.31 × 10^−05^	8.33 × 10^−05^	9.11 × 10^−05^
Bi-213	7.95 × 10^−05^	4.12 × 10^−05^	4.12 × 10^−05^	5.38 × 10^−05^
(Po-213)	1.61 × 10^−03^	4.92 × 10^−04^	4.93 × 10^−04^	8.36 × 10^−04^
(T1-209)	1.03 × 10^−04^	7.94 × 10^−05^	7.95 × 10^−05^	8.84 × 10^−05^
(Pb-209)	2.87 × 10^−05^	1.37 × 10^−05^	1.37 × 10^−05^	1.85 × 10^−05^
Ra-223	1.18 × 10^−03^	3.15 × 10^−04^	3.15 × 10^−04^	5.83 × 10^−04^
(Rn-219)	1.35 × 10^−03^	3.83 × 10^−04^	3.84 × 10^−04^	6.80 × 10^−04^
(Po-215)	1.46 × 10^−03^	4.22 × 10^−04^	4.22 × 10^−04^	7.41 × 10^−04^
(Pb-211)	5.55 × 10^−05^	3.48 × 10^−05^	3.49 × 10^−05^	4.20 × 10^−05^
(Bi-211)	1.31 × 10^−03^	3.70 × 10^−04^	3.71 × 10^−04^	6.60 × 10^−04^
(T1-207)	5.85 × 10^−05^	3.72 × 10^−05^	3.73 × 10^−05^	4.47 × 10^−05^
(Po-211)	1.47 × 10^−03^	4.25 × 10^−04^	4.26 × 10^−04^	7.47 × 10^−04^
Ac-225	1.19 × 10^−03^	3.18 × 10^−04^	3.19 × 10^−04^	5.87 × 10^−04^
(Fr-221)	1.27 × 10^−03^	3.54 × 10^−04^	3.54 × 10^−04^	6.36 × 10^−04^
(At-217)	1.40 × 10^−03^	4.02 × 10^−04^	4.02 × 10^−04^	7.10 × 10^−04^
Th-227	1.22 × 10^−03^	3.29 × 10^−04^	3.30 × 10^−04^	6.03 × 10^−04^
*Beta and positron emitters*				
Sr-89	6.65 × 10^−05^	4.43 × 10^−05^	4.44 × 10^−05^	5.23 × 10^−05^
Y-90	9.54 × 10^−05^	7.23 × 10^−05^	7.24 × 10^−05^	8.14 × 10^−05^
I-124	3.59 × 10^−05^	3.03 × 10^−05^	3.03 × 10^−05^	3.27 × 10^−05^
I-131	3.35 × 10^−05^	1.88 × 10^−05^	1.88 × 10^−05^	2.37 × 10^−05^
Sm-153	4.33 × 10^−05^	1.94 × 10^−05^	1.94 × 10^−05^	2.70 × 10^−05^
Ho-166	8.05 × 10^−05^	5.32 × 10^−05^	5.32 × 10^−05^	6.29 × 10^−05^
Lu-177	2.43 × 10^−05^	1.02 × 10^−05^	1.02 × 10^−05^	1.47 × 10^−05^
Re-186	4.44 × 10^−05^	2.47 × 10^−05^	2.47 × 10^−05^	3.13 × 10^−05^
Re-188	8.55 × 10^−05^	6.07 × 10^−05^	6.08 × 10^−05^	7.00 × 10^−05^
*Auger electron emitters*				
Pd-103	3.11 × 10^−06^	1.08 × 10^−06^	1.05 × 10^−06^	1.69 × 10^−06^
In-111	1.32 × 10^−05^	8.93 × 10^−06^	8.91 × 10^−06^	1.03 × 10^−05^
Sn-117 m	3.06 × 10^−05^	1.32 × 10^−05^	1.31 × 10^−05^	1.86 × 10^−05^
I-123	9.28 × 10^−06^	5.01 × 10^−06^	4.97 × 10^−06^	6.35 × 10^−06^
I-125	8.70 × 10^−06^	2.68 × 10^−06^	2.60 × 10^−06^	4.50 × 10^−06^
Pt-193 m	2.80 × 10^−05^	7.76 × 10^−06^	7.77 × 10^−06^	1.41 × 10^−05^
Pt-195 m	4.22 × 10^−05^	1.07 × 10^−05^	1.07 × 10^−05^	2.05 × 10^−05^

**Table 9.** S-values for diagnostic radionuclides for the adult female.

Radionuclide	*S*-values (mGy/MBq-s)			
	*S* (LB ← LB)	*S* (LB ← LP)	*S* (LP ← LB)	*S* (LP ← LP)

*SPECT radionuclides*				
Ga-67	1.04 × 10^−05^	4.50 × 10^−06^	4.49 × 10^−06^	6.37 × 10^−06^
Tc-99 m	5.05 × 10^−06^	2.82 × 10^−06^	2.81 × 10^−06^	3.53 × 10^−06^
In-111	1.32 × 10^−05^	8.93 × 10^−06^	8.91 × 10^−06^	1.03 × 10^−05^
I-123	9.28 × 10^−06^	5.01 × 10^−06^	4.97 × 10^−06^	6.35 × 10^−06^
*PET radionuclides*				
C-11	6.21 × 10^−05^	4.31 × 10^−05^	4.32 × 10^−05^	4.99 × 10^−05^
N-13	7.24 × 10^−05^	5.17 × 10^−05^	5.18 × 10^−05^	5.92 × 10^−05^
O-15	9.36 × 10^−05^	7.13 × 10^−05^	7.14 × 10^−05^	8.00 × 10^−05^
F-18	4.68 × 10^−05^	3.14 × 10^−05^	3.15 × 10^−05^	3.68 × 10^−05^
Ga-68	9.12 × 10^−05^	7.08 × 10^−05^	7.09 × 10^−05^	7.89 × 10^−05^
Rb-82	1.46 × 10^−04^	1.26 × 10^−04^	1.26 × 10^−04^	1.36 × 10^−04^

## Data Availability

The data of the study will be made available upon request of the authors. PHITS radiation transport files for all Monte Carlo radiation transport simulations conducted herein will be made available at a publicly accessible GitHub repository. All data that support the findings of this study are included within the article (and any supplementary information files).

## Footnotes

7 cgal.org.

8 https://bioart.niaid.nih.gov/.

9 https://physics.nist.gov/PhysRefData/Star/Text/ASTAR.html.

10 https://physics.nist.gov/PhysRefData/Star/Text/ESTAR.htm.
